# A deep learning framework for *in silico* screening of anticancer drugs at the single-cell level

**DOI:** 10.1093/nsr/nwae451

**Published:** 2024-12-10

**Authors:** Peijing Zhang, Xueyi Wang, Xufeng Cen, Qi Zhang, Yuting Fu, Yuqing Mei, Xinru Wang, Renying Wang, Jingjing Wang, Hongwei Ouyang, Tingbo Liang, Hongguang Xia, Xiaoping Han, Guoji Guo

**Affiliations:** Bone Marrow Transplantation Center of the First Affiliated Hospital, and Center for Stem Cell and Regenerative Medicine, Zhejiang University School of Medicine, Hangzhou 310000, China; Liangzhu Laboratory, Zhejiang University, Hangzhou 311121, China; Dr. Li Dak Sum & Yip Yio Chin Center for Stem Cell and Regenerative Medicine, Zhejiang University, Hangzhou 310058, China; Bone Marrow Transplantation Center of the First Affiliated Hospital, and Center for Stem Cell and Regenerative Medicine, Zhejiang University School of Medicine, Hangzhou 310000, China; Liangzhu Laboratory, Zhejiang University, Hangzhou 311121, China; Research Center of Clinical Pharmacy of the First Affiliated Hospital, Zhejiang University School of Medicine, Hangzhou 310003, China; Department of Hepatobiliary and Pancreatic Surgery, the First Affiliated Hospital, Zhejiang University School of Medicine, Hangzhou 310003, China; MOE Joint International Research Laboratory of Pancreatic Diseases, Hangzhou 310003, China; Zhejiang Provincial Key Laboratory of Pancreatic Disease, the First Affiliated Hospital, Zhejiang University School of Medicine, Hangzhou 310003, China; Zhejiang University Cancer Center, Hangzhou 310058, China; Bone Marrow Transplantation Center of the First Affiliated Hospital, and Center for Stem Cell and Regenerative Medicine, Zhejiang University School of Medicine, Hangzhou 310000, China; Bone Marrow Transplantation Center of the First Affiliated Hospital, and Center for Stem Cell and Regenerative Medicine, Zhejiang University School of Medicine, Hangzhou 310000, China; Bone Marrow Transplantation Center of the First Affiliated Hospital, and Center for Stem Cell and Regenerative Medicine, Zhejiang University School of Medicine, Hangzhou 310000, China; Bone Marrow Transplantation Center of the First Affiliated Hospital, and Center for Stem Cell and Regenerative Medicine, Zhejiang University School of Medicine, Hangzhou 310000, China; Bone Marrow Transplantation Center of the First Affiliated Hospital, and Center for Stem Cell and Regenerative Medicine, Zhejiang University School of Medicine, Hangzhou 310000, China; Liangzhu Laboratory, Zhejiang University, Hangzhou 311121, China; Liangzhu Laboratory, Zhejiang University, Hangzhou 311121, China; Dr. Li Dak Sum & Yip Yio Chin Center for Stem Cell and Regenerative Medicine, Zhejiang University, Hangzhou 310058, China; Department of Hepatobiliary and Pancreatic Surgery, the First Affiliated Hospital, Zhejiang University School of Medicine, Hangzhou 310003, China; MOE Joint International Research Laboratory of Pancreatic Diseases, Hangzhou 310003, China; Zhejiang Provincial Key Laboratory of Pancreatic Disease, the First Affiliated Hospital, Zhejiang University School of Medicine, Hangzhou 310003, China; Zhejiang University Cancer Center, Hangzhou 310058, China; Liangzhu Laboratory, Zhejiang University, Hangzhou 311121, China; Research Center of Clinical Pharmacy of the First Affiliated Hospital, Zhejiang University School of Medicine, Hangzhou 310003, China; Department of Biochemistry, Zhejiang University School of Medicine, Hangzhou 310058, China; Bone Marrow Transplantation Center of the First Affiliated Hospital, and Center for Stem Cell and Regenerative Medicine, Zhejiang University School of Medicine, Hangzhou 310000, China; Zhejiang Key Laboratory of Multi-omics Precision Diagnosis and Treatment of Liver Diseases, Sir Run Run Shaw Hospital, Zhejiang University School of Medicine, Hangzhou 310000, China; Bone Marrow Transplantation Center of the First Affiliated Hospital, and Center for Stem Cell and Regenerative Medicine, Zhejiang University School of Medicine, Hangzhou 310000, China; Liangzhu Laboratory, Zhejiang University, Hangzhou 311121, China; Dr. Li Dak Sum & Yip Yio Chin Center for Stem Cell and Regenerative Medicine, Zhejiang University, Hangzhou 310058, China; Institute of Hematology, Zhejiang University, Hangzhou 310000, China

**Keywords:** targeted therapy, scRNA-seq, machine learning, pan-cancer, drug screening

## Abstract

Tumor heterogeneity plays a pivotal role in tumor progression and resistance to clinical treatment. Single-cell RNA sequencing (scRNA-seq) enables us to explore heterogeneity within a cell population and identify rare cell types, thereby improving our design of targeted therapeutic strategies. Here, we use a pan-cancer and pan-tissue single-cell transcriptional landscape to reveal heterogeneous expression patterns within malignant cells, precancerous cells, as well as cancer-associated stromal and endothelial cells. We introduce a deep learning framework named Shennong for *in silico* screening of anticancer drugs for targeting each of the landscape cell clusters. Utilizing Shennong, we could predict individual cell responses to pharmacologic compounds, evaluate drug candidates’ tissue damaging effects, and investigate their corresponding action mechanisms. Prioritized compounds in Shennong's prediction results include FDA-approved drugs currently undergoing clinical trials for new indications, as well as drug candidates reporting anti-tumor activity. Furthermore, the tissue damaging effect prediction aligns with documented injuries and terminated discovery events. This robust and explainable framework has the potential to accelerate the drug discovery process and enhance the accuracy and efficiency of drug screening.

## INTRODUCTION

The extensive heterogeneity of tumors plays a pivotal role in cancer progression and resistance to clinical treatment [1]. The dynamic interplay between cancer cells and their microenvironment forms an intricate ecosystem [2]. Unraveling the mechanisms underpinning tumor progression is crucial for precise diagnosis, prognosis, and therapy. Pan-cancer analyses, utilizing data across various cancer types, could reveal common underlying drivers of oncogenesis and progression. This illuminates common genomic alterations, signaling pathways, and cellular processes across diverse cancer types [3,4]. A comprehensive understanding of the pan-cancer landscape assists in guiding therapeutic strategies, discovering novel targeted treatment cancer types, and advancing personalized medicine [4,5].

High-throughput single-cell RNA-sequencing (scRNA-seq) enables the capture of unbiased individual cell transcriptomes [6,7]. It allows us to identify intra-tumor heterogeneity and characterize rare cancer cells hidden in traditional bulk analyses [4]. scRNA-seq reveals the multifaceted interactions between cancer cells and their microenvironment, including the roles of non-malignant and immune cells, as well as the extracellular matrix, which significantly influences tumor initiation and progression. These insights can uncover mechanisms of resistance and lead to the discovery of novel diagnostic and prognostic markers. Tracking development and responses of individual cancer cells over time could enhance our understanding of tumor dynamics and inform therapeutic strategies, ultimately improving cancer treatment precision and efficacy [2].

Recent pan-cancer studies based on scRNA-seq have focused primarily on tumor microenvironments (TMEs), especially immune cells, while often neglecting intra-tumor heterogeneity and complex inter-component interactions.

Artificial intelligence, specifically machine learning, has been playing important roles in cancer research and pharmaceutical innovation [8–10]. It can integrate extensive cancer datasets, decode the complex biological systems, and identify common and unique tumor patterns. In drug discovery, machine learning enhances our understanding of drug–target interactions, improves therapeutic strategies, enriches assessments of drug effectiveness and potential side effects, and accelerates the identification of new drug candidates [8,10]. Large perturbation datasets are beneficial tools to explore how human diseases and their gene expression profiles are affected by different kinds of chemical compounds, including potential drugs [11].

Several computational tools have emerged in drug response predictions. For example, scRANK ranks cell types based on their gene network activity in response to drugs, but its reliance on prior knowledge limits its use for compounds with unclear targets [12]. BeyondCell identifies cellular contexts that influence drug sensitivity but lacks interpretability and specific drug targets, complicating clinical application. scDEAL employs a deep transfer learning to model drug responses by integrating bulk and single-cell RNA-seq data, though harmonizing these data types can be challenging and may introduce biases. These advancements underscore the potential of machine learning to enhance drug discovery and personalize cancer treatment.

**Figure 1.**
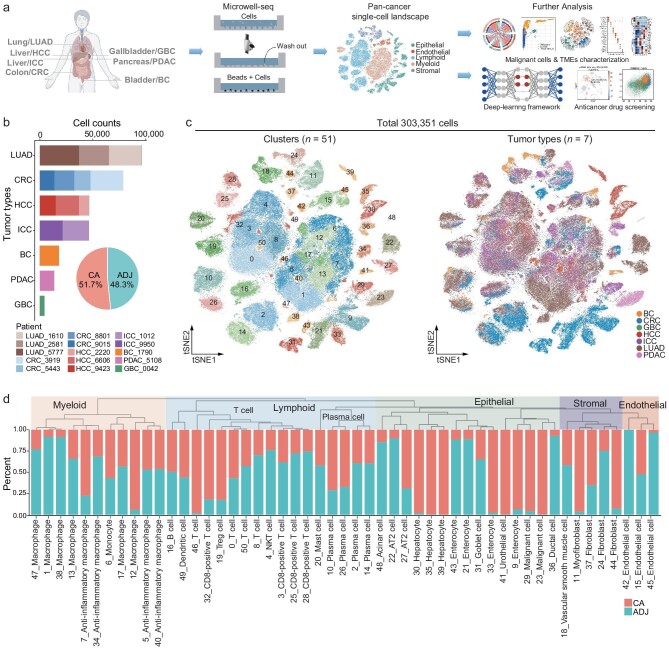
The pan-cancer cell landscape was constructed using Microwell-seq. (a) Overview of scRNA-seq experiments and bioinformatics workflow. Created with BioRender.com. (b) Stacked bar chart showing the number of analyzed cells from each tumor type and each patient, and pie chart showing the percentage of analyzed cells in tumor (CA) and adjacent (ADJ) tissues. (c) *t*-SNE visualization of 303 351 single cells from the pan-cancer landscape, colored by cluster identity (*n* = 51) and tumor type (*n* = 7). (d) Hierarchical clustering tree (top) showing the similarity among 51 cell clusters, and histogram (bottom) showing the percentage of tissue source for each cell cluster.

In this study, we profiled the pan-cancer single-cell transcriptional landscape using Microwell-seq, with 303 351 cells across 7 common solid cancer types (Fig. 1a). Integrating public scRNA-seq data of corresponding normal tissues, we characterized the heterogeneous expression patterns within malignant and precancerous cells, as well as cancer-associated stromal and endothelial cells. We found that precancerous cells not only originate from adjacent tissue but undergo molecular alterations associated with nearby tumor tissue, reflecting an intermediate state from normal to tumor.

The pan-cancer single-cell landscape offers valuable resources for anticancer drug development. By utilizing this detailed cellular atlas, we aim to enhance the design of targeted therapeutic strategies that aligns drug responses with specific cellular contexts. We introduced Shennong, a deep learning framework that allows large-scale screening of anticancer drugs for targeting each of the landscape cell clusters. By leveraging pan-cancer data to predict single cells’ responses to pharmacological perturbations, we further screened both broad-spectrum and cancer-specific drug candidates, identified new indications for marketed drugs, evaluated drug candidates’ tissue damaging effects, and explored their corresponding action mechanisms.

Prioritized compounds in Shennong's prediction results include FDA-approved drugs undergoing clinical trials for new indications, such as azacitidine and irinotecan, along with drug candidates like parbendazole. Tissue damaging effect predictions were consistent with reported injuries or terminated discovery events, like vemurafenib, lopinavir, and GSK-690693. Our framework was robust and highly interpretable, revealing hidden mechanisms of action and efficiently predicting pharmaceutical effects on specific cell types.

Overall, we profiled the pan-cancer landscape, explored its heterogeneous expression patterns, and proposed the Shennong framework, a single-cell level drug screening framework to enhance the accuracy and efficiency of drug screening, expediting drug repurposing and discovery. The pan-cancer landscape and training and prediction results of the Shennong framework are available on our website (http://bis.zju.edu.cn/shennong/index.html).

## RESULTS

### Construction of the pan-cancer single-cell landscape using Microwell-seq

To investigate cell type diversity across different cancer types, we compiled a pan-cancer single-cell landscape using Microwell-seq. Tumor (CA) and adjacent (ADJ) tissues were dissociated to single-cell suspension for scRNA-seq profiles without initial sorting. After quality control and filtering, our landscape consisted of 303 351 cells from 15 patients spanning 7 tumor types, with 51.7% of cells originating from tumor tissues (Fig. 1b and Tables S1–S5). Our tumor collection included lung adenocarcinoma (LUAD), colorectal cancer (CRC), hepatocellular carcinoma (HCC), intrahepatic cholangiocarcinoma (ICC), bladder cancer (BC), pancreatic ductal adenocarcinoma (PDAC), and gallbladder cancer (GBC), containing the highest morbidity and mortality tumor types in China.

Using unsupervised clustering, we identified 51 major cell clusters (Fig. 1c, Fig. S1a, b and Table S6). These clusters were classified based on cell type-specific markers into 5 main lineages: lymphoid (*CD3D, CD3E, CD79A, JCHAIN*), myeloid (*CD68, CD14*), stromal (*DCN, COL1A1, ACTA2*), endothelial (*VWF, PLVAP*), and epithelial (*EPCAM, KRT18*) (Fig. S1c–e and Table S7). The cell-type hierarchy tree showed that cell clusters from the same cell lineage tended to converge together beyond the tissue type (Fig. 1d and Fig. S1f).

Upon observing the composition of each cluster, we found that nearly all epithelial cells displayed tissue type-specific characteristics, with contributions from multiple patients, while some clusters showed patient-specific patterns (Fig. S1g). For example, C9, C21, and C43 were multi-patient contributed enterocytes, while C33 was primarily contributed by patient CRC_9015. In terms of hepatocytes, each cluster (C30, C35, and C39) was predominantly contributed by a single patient. Interestingly, C36 was identified as ductal cells with contributions from multiple tumor types, consisting of cells from HCC, ICC, and PDAC patients. However, no significant differences in the TME were observed, regardless of malignant status and tissue type (Fig. 1d and Fig. S1f, g). Stromal, endothelial, and immune clusters derived from different tumor types and patients were well integrated, respectively. Clusters C11 and C24 were myofibroblast/fibroblast, and C15 and C42 were endothelial cells. The lymphoid lineage mainly included T cells, B cells, and plasma cells, while the myeloid lineage primarily included macrophages, monocytes, and dendritic cells. Notably, C46 had T cells derived from multiple HCC patients, while C1, C38, and C47 mainly originated from LUAD patients, particularly C47. These results indicated that, although sharing many commonalities in the pan-cancer landscape, cell types exhibited distinct features across tumor types and individual patients. This underscores the significance of considering both tumor types and patient characteristics in cancer research and treatment strategies.

### Heterogeneous expression patterns within malignant and precancerous cells

**Figure 2.**
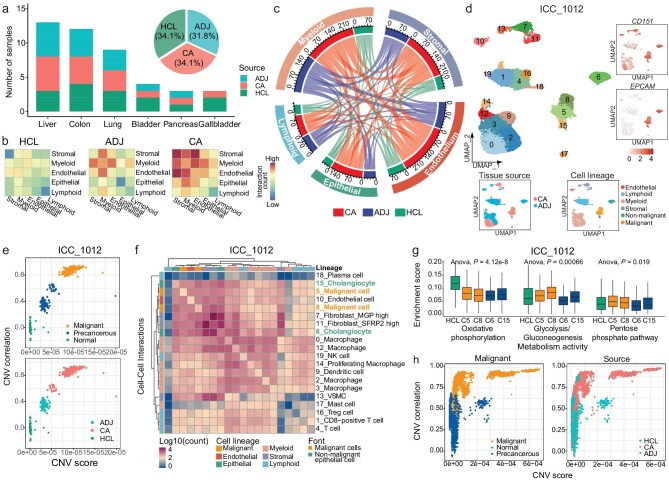
Identification of malignant and precancerous cells via single-sample analyses. (a) Stacked bar chart showing the number of samples from each tissue type and source in the pan-cancer landscape and HCL, and pie chart showing the percentage of analyzed samples in CA, ADJ, and normal (HCL) tissues. (b) The mutual cell interaction among 5 main cell lineages in TMEs from different tissue sources. (c) Interactions between 5 main cell lineages in TME. The length of arcs represents the predicted interaction counts. (d) UMAP visualization of 24 628 single cells from patient ICC_1012 (top left), colored by *CD151* and *EPCAM* enrichment (top right), tissue sources (bottom left), and cell lineages (bottom right). (e) Malignant type classification (top) and tissue source distribution (bottom) of inferred CNV scores (x-axis) and CNV correlations (y-axis) for all epithelial cells of patient ICC_1012. (f) The cell interactions between cell clusters for patient ICC_1012. Malignant cell types are colored orange and non-malignant epithelial cell types are colored green. (g) Boxplot showing enrichment scores of ‘oxidative phosphorylation’, ‘glycolysis/gluconeogenesis’ and ‘pentose phosphate pathway’ metabolism pathways in epithelial clusters of corresponding normal tissue and patient ICC_1012. (h) Malignant type classification (left) and tissue source distribution (right) of inferred CNV scores (x-axis) and CNV correlations (y-axis) for all epithelial cells in the pan-cancer landscape.

Understanding intra-tumor heterogeneity is important to decipher cancer progression and improving treatment efficacy. By integrating scRNA-seq data of corresponding normal tissues from our previous work, Human Cell Landscape (HCL) [7], we investigated the cellular heterogeneity of tumor cells and their TMEs. All scRNA-seq data were generated using the same platform Microwell-seq, in other words, this excludes possible technology-induced bias. Our merged dataset included 388 646 cells, with approximately one-third originating from CA, ADJ, and normal tissue samples (Fig. 2a and Fig. S2a–c). Cells from HCL accounted for 22% of the dataset. Cell–cell communication analysis revealed that interactions significantly increased (*p* < 0.05) in the order of normal, ADJ, and CA tissues (Fig. 2b, c and Fig. S2d), suggesting that adjacent tissue might be in transitional states between normal and tumor tissues.

Accurate definition of malignant cells is essential for characterizing tumor heterogeneity patterns. Given that simply classifying cells as epithelial is inadequate for identifying malignant cells in tumor tissues [13], we simultaneously employed three approaches to accurately identify malignant cells and annotate other cells (Fig. 2d, e and Figs S3, S4; see Materials and Methods). Using patient ICC_1012 as an example, we first assigned cells to distinct cell types and identified significantly highly expression genes in different epithelial clusters (Fig. 2d). Then, we inferred copy number variation (CNV) scores in these epithelial clusters using normal colon epithelial cells from HCL as references (Fig. 2e). Third, potential malignant cells forming separate clusters in dimensionality reduction. Compared to non-malignant epithelial cells, malignant cells exhibited higher CNV scores and formed separate clusters in ICC_1012, despite both *EPCAM* and *CD151* [14] being overexpressed in these epithelial cells (Fig. 2d, e).

Interestingly, almost all non-malignant epithelial cells showed high expressed of cancer-related genes and exhibited borderline CNV scores. For example, clusters C18 in BC_1790, C6 and C15 in ICC_1012, and C4, C11, and C20 in PDAC_5108 (Fig. 2d, e and Figs S3, S4). Analyses of cell–-cell communication and metabolic activity indicated that these cells resembled malignant cells (Fig. 2f, g, Fig. S5a–c, and Tables S8–S10), showing close interactions with TMEs and a downregulation of oxidative phosphorylation, alongside an upregulation of glycolysis and gluconeogenesis. The majority of these cells originated from adjacent tissues and did not cluster with malignant cells. Notably, non-malignant epithelial cells originating from tumor tissues displayed intermediate CNV scores, such as C15 in ICC_1012, C13 and C22 in LUAD_1610, C16 and C20 in LUAD_5777, and C20 in PDAC_5108 (Fig. 2d, e and Figs S3, S4). The C20 cluster in PDAC_5108 might be undergoing epithelial-to-mesenchymal transition (EMT), as indicated by the highest EMT scores (Fig. S5d). These observations were also supported by published scRNA-seq tumor datasets [15,16] from the 10x Chromium platform (Fig. S5e–j), using corresponding tissues from *Tabula Sapiens* as healthy normal tissues.

Therefore, our CNV analyses, combined with tumor marker gene expression, cell–cell communication, and metabolic activity assessments, indicate that non-malignant epithelial cells resembling malignant cells might signify an early stage of cancerization [17], representing a transitional state from normal to tumor tissue. We termed these cells ‘precancerous cells’ in our study, distinguishing them from both normal epithelial and malignant cells. Overall, we identified 34 926 high-confidence malignant cells and 23 034 non-malignant (precancerous) cells across the pan-cancer landscape (Fig. 2h).

**Figure 3.**
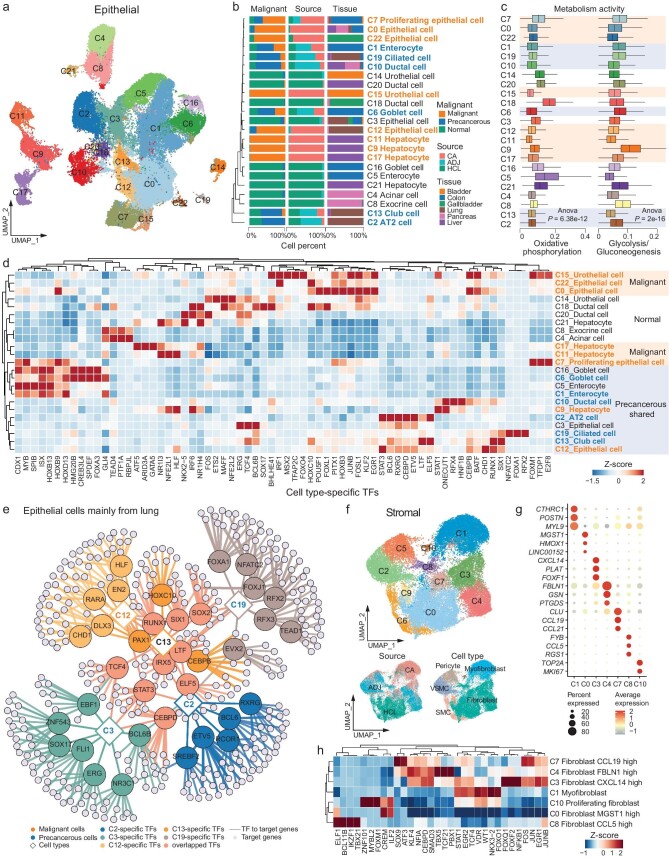
Profiling malignant and tumor-associated stromal cells via pan-cancer analyses. (a) UMAP visualization of clusters (*n* = 23) for all epithelial cells from the pan-cancer landscape and HCL. (b) Bar plot showing the percentage of malignant type classification (left), tissue source (middle), and tissue type (right) for each epithelial cluster. (c) Boxplot showing enrichment scores for the ‘oxidative phosphorylation’ and ‘glycolysis/gluconeogenesis’ metabolism pathways in all epithelial clusters. (d) Heatmap showing cell type-specific TFs detected by SCENIC analysis. Malignant cell types are colored orange and precancerous cell types are colored blue. (e) Gene regulatory networks showing relationships between TFs and their target genes for epithelial cell clusters mainly originating in the lung. (f) UMAP visualization of all stromal cells from the pan-cancer landscape and HCL, colored by clusters (*n* = 11, top), tissue source (bottom left), and main cell type (bottom right). (g) Dot plots showing scaled average expression levels of cell type-specific markers in fibroblast/myofibroblast clusters. (h) Heatmap showing cell type-specific TFs detected in fibroblast/myofibroblast clusters by SCENIC analysis.

Next, we re-clustered all malignant, precancerous, and normal epithelial cells from each patient in our pan-cancer landscape and HCL. Malignant, precancerous, and normal epithelial cells accounted for 38.9%, 25.6%, and 35.5%, respectively. We identified 23 clusters (C0–C22) through unsupervised clustering, annotating them according to cell type-specific markers (Fig. 3a, b, Fig. S6a–c, and Table S11). Clusters dominated by normal cells (C3, C5, C20, C21, C16) contained cells from multiple donors and tissues (Fig. 3b and Fig. S6a, b), indicating minimal batch effects. However, clusters dominated by malignant cells (C9, C11, C17, C12, C15, C22) were primarily composed of cells from single patients, displaying extensive inter-tumor heterogeneity. Precancerous cells clustered with normal or malignant cells, forming independent clusters from specific tissues (C2, C6, C10, C19) or structures (C13), reflecting diverse intermediary malignant states. Unsurprisingly, epithelial cells were involved in distinct pathways (Fig. S6d). Hallmark and metabolism analyses revealed downregulation of oxidative phosphorylation in malignant and precancerous clusters, contrasted by increased glycolysis and mTORC1 signaling (Fig. 3c and Fig. S6d), consistent with typical observations in cancer cell bioenergetics [18,19]. Meanwhile, cell proliferation-related gene sets were enriched in malignant clusters, including E2F targets, G2M checkpoint, and MYC targets. Pathways known to be involved in tumor initiation and progression [20], such as Wnt, Notch, and TGF-β, were also enriched in malignant and precancerous clusters. We then investigated the regulatory patterns in tumor development, focusing on potential regulon involvement in malignant and precancerous clusters (Fig. 3d). Malignant and normal clusters showed distinct regulatory patterns, while precancerous clusters displayed shared patterns with both. Regulators associated with tumorigenesis and tumor progression, such as EGR1, FOSL, BATF, and HOXB3, were enriched in malignant clusters [21,22]. In contrast, normal clusters were enriched in regulators related to cell development and differentiation, such as TCF4. Precancerous clusters showed enrichment in tumor progression-related regulons, including transcription factors (TFs) linked to tumor proliferation and cell cycle progression (STAT1, BCL6, ETV5) and potential tumor suppressors (ELF5, LTF, RXRG, CEBPD), as well as EMT-related regulons. In the cell clusters collected from the lung (C2, C3, C12, C13, and C19), shared TFs among malignant and precancerous cells included LTF, IRX5, SIX1, and RUNX1, associated with tumor invasion and metastasis. The potential tumor suppressor ELF5 was found only in precancerous clusters (Fig. 3e). The EMT regulators, SOX2 and TCF4, were shared in both normal and precancerous clusters (Fig. 3e). The distinct regulatory patterns observed in malignant, precancerous, and normal clusters highlight the intermediate states of precancerous cells between tumor and healthy normal tissue, emphasizing the importance of precise precancerous cell identification.

### Characterization of cancer-associated fibroblasts and endothelial cells through pan-cancer analyses

Similar to malignant cells, the heterogeneity of TME composites is an important part of tumors, whose interaction with malignant cells significantly affects tumor progression and metastasis. Our analysis of cell–cell communication revealed that crosstalk among stromal, endothelial, and myeloid cells predominated in the TME with stromal cells interacting most with the other components across tumor and adjacent samples, regardless of tissue type (all *p*  <  0.05) (Fig. 2c and Fig. S2d). This indicated the important role of communication among TME components.

To characterize stromal cells in the TME, we re-clustered all stromal cells from each patient in the pan-cancer landscape and HCL. We found a clear separation between normal tissues and tumor-associated tissues (Fig. 3f and Fig. S7a). Different from epithelial cells, the CNV scores of stromal cells in tumor and adjacent tissues were not significantly different, but were significantly higher than those of normal stromal cells from HCL (Fig. S7b). This lack of significant difference indicated that stromal cells in adjacent tissues may exhibit characteristics similar to those in tumors, likely due to the complex interactions within the TME [23].

These 35 661 stromal cells could be divided into 11 distinct clusters (Fig. 3f, g, Fig. S7c, d, and Table S12). C1 (myofibroblast) and C0 (fibroblast_MGST1 high) primarily originated from tumor tissues and normal tissues, respectively. Other fibroblasts (C3, C4, C7, C8, and C10) included cells from both tumor and adjacent tissues, expressing markers associated with cancer-associated fibroblasts (CAFs) (Fig. 3g). C3 was identified as inflammatory CAF, marked by high expression of cytokines and chemokines, such as *CXCL14, CXCL12, PLAT*, and *FOXF1. CXCL12* and *CXCL14* have previously been used as CAF markers related to immune and inflammatory regulation [24,25]. C4 expressed *FBLN1*, a classical CAF marker, while C7, a rare reticular-like CAF cluster, showed strong expression of *CCL19* and *CCL21*, markers of reticular fibroblasts in lymphoid tissues that contribute to the homing of naïve T cells [26].

Gene Set Enrichment Analysis (GSEA) indicated that myofibroblasts had significantly higher EMT scores compared to other stromal cells, CAFs (C3, C4, C7, and C8), and normal fibroblasts (Fig. S7d), indicating that the general dedifferentiated process of myofibroblast along CAF activation [27]. This also suggested that epithelial transdifferentiation might be a possible major source of myofibroblast and provided new potential targets for therapeutic strategies. Hallmark analyses showed downregulation of oxidative phosphorylation and upregulation of glycolytic signaling in CAFs, with enrichment in tumorigenesis and progression-related pathways (Fig. S7e), which was consistent with the results in malignant and precancerous cells (Fig. 3b and Fig. S6d). Additionally, cell-cycle and cell proliferation-related gene sets were enriched in CAFs (C1, C3, C4, C7, and C8), as well as pericytes (C5) and vascular smooth muscle cells (VSMC, C2), all of which originated from tumor and adjacent tissues. When focusing on regulatory patterns, we found that CAFs displayed distinct cell type-specific TFs alongside shared TFs widely involved in carcinogenesis (Fig. 3h), including various tumor promoters or tumor suppressors, such as KLF4, NFIA, TCF21, NFKB1, and EGR1. These suggested that CAFs may have different differentiated states and effects on the TME, while sharing similar regulatory patterns.

We then obtained 17 564 endothelial cells from pan-cancer landscape and HCL (Fig. S8a–c), which were divided into 10 cell clusters (Table S13). Besides endothelial tip cells (*COL4A1*), we identified endothelial subtypes from traditional vascular beds, including arterial (*SEMA3G*), capillary (*CA4, PRX*), venous (*ACKR1*), and lymphatic endothelial cells (*CCL21, PROX1*) [28] (Fig. S8b). We distinguished specialized sinusoidal endothelial cells (*FCN2*) from normal and adjacent tissues, which play roles in homeostatic, filtration, endocytic, and immunological functions [29].

Our analysis revealed downregulation of oxidative phosphorylation in cancer-associated endothelial cells (C0, C1, C4, C5, and C6), primarily originated from tumor and adjacent tissues (Fig. S8d). In these clusters, gene sets related to cell proliferation and tumor initiation were enriched, including pathways such as E2F targets, G2M checkpoint, MYC targets v2, Wnt, PI3K/AKT/mTOR, Notch, TGF-β, and Hedgehog signaling (Fig. S8d). The cluster with the highest cell composition of tumor tissue, C0 (endothelial tip cells), showed significant enrichment in processes regulating angiogenesis (Fig. S8e), suggesting that cancer-associated endothelial cells might contribute to tumor remodeling and progression through angiogenesis stimulation [30]. Additionally, sinusoidal endothelial cells from adjacent tissues (C4) displayed significant enrichment in processes related to the ERK1 and ERK2 cascade, epithelial cell migration and proliferation, and cell-substrate adhesion compared to normal sinusoidal endothelial cells (C3) (Fig. S8f).

These results once again emphasized the different states of cells in adjacent and normal tissue. Understanding the differences between tumor-adjacent tissues and healthy normal tissues helps reveal the mechanisms of tumor development and communication with surrounding tissues.

### Interpretable single-cell level drug perturbation prediction using the deep learning framework

Construction of the pan-cancer single-cell landscape and characterization of heterogeneous expression patterns in malignant cells and TME allowed us to observe the different states of both tumor cells and normal cells. This helped to explore potential therapeutic pathways and potential targets for various cancer types. Machine learning methods combined with high-throughput and cost-effective perturbation datasets allow efficient exploration of how different classes of compounds, especially drug candidates, affect human diseases and their gene expression profiles. This integration improves the design of targeted therapies. Therefore, we proposed a deep learning framework called Shennong. This framework could describe the individual cancer cells’ responses to pharmacologic perturbations, screen potential anticancer drug targets, and evaluate potential tissue damaging effects. Single-cell level prediction with machine learning methods could accelerate the drug discovery process and enhance the accuracy and efficiency of drug screening.

**Figure 4.**
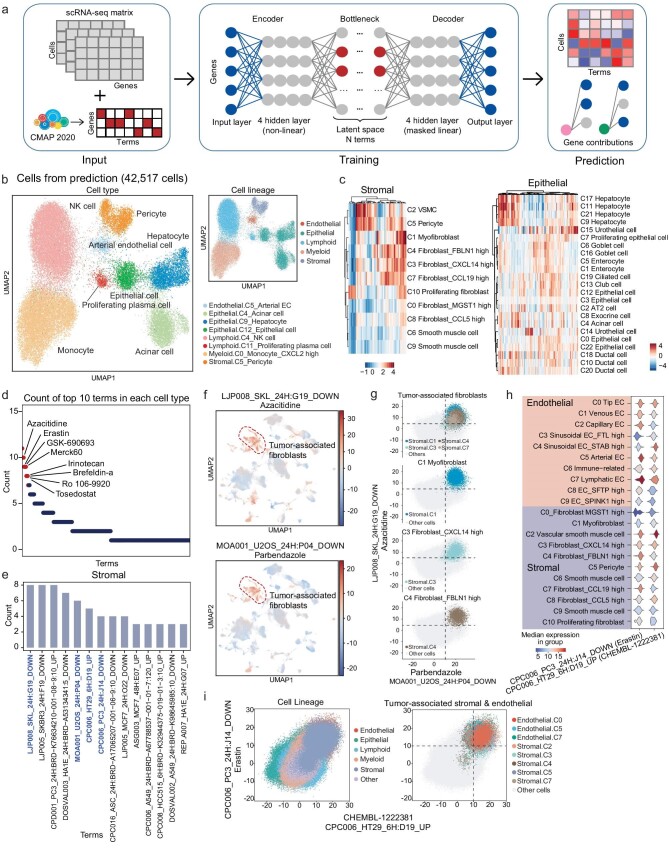
Interpretable single-cell level drug perturbation prediction using Shennong. (a) Workflow of the Shennong framework. The framework employs an interpretable conditional variational autoencoder, trained on perturbation data matrix and scRNA-seq count matrix for each cell to encode a set of significant features representing terms. The terms are pruned and enriched by the framework using a group lasso and gene-level sparsity regularization which was then fed into a linear decoder. The framework was interpretable by calculating the influence term score matrix of specific terms for each cell and the contribution of individual genes in each term. (b) UMAP representation of the prediction set (*n* = 42 517 cells) embedded in latent space extracted from the framework, colored by cell type (left) and cell lineage (right). (c) Heatmaps showing the scaled influence term scores of the top 10 significantly differential terms (columns) in stromal cells (left) and epithelial cells (right). (d) Dot plot showing counts of the top 10 significantly differential terms in each cell type in the single-lineage analyses with the compounds corresponding to the terms labeled. (e) Bar plot showing counts of the top 10 significantly differential terms in each cell type in stromal lineage. Terms that are further analyzed are colored blue. (f) UMAP representation of the influence term scores of all cells for terms LJP008_SKL_24H: G19_DOWN (top) and MOA001_U2OS_24H: P04_DOWN (bottom), corresponding to the FDA-approved drugs azacitidine and palbendazole, respectively. (g) Visualization of selected cell types (CAFs) in the context of the terms mentioned in (f). Each dot shows the influence term scores of each cell. (h) Violin plot showing the influence term scores for terms mentioned in (e) for all cell types in the stromal and endothelial lineages. (i) Visualization of cell lineages (top) and tumor-associated stromal or endothelial cell types (bottom) in the context of the terms mentioned in (e).

The Shennong framework consists of three main stages (Fig. 4a; see Materials and Methods). In brief, we first integrated the scRNA-seq count matrix with preprocessed perturbation data to obtain the perturbation changes at the cell level. We created a binary matrix representing genetic changes associated with specific gene sets (referred to as terms) linked to various compounds. Each term captures a unique combination of perturbations relevant to experimental conditions. This matrix was extracted from high-confidence signatures of CMap (2020 version) [11], which contains ∼8 billion gene expression profiles from over 240 human cell lines exposed to >39 000 compounds. It retains features that were significantly differentially expressed within each term (Fig. S9a). Next, we used scRNA-seq gene expression profiles and condition labels for each cell to encode a set of terms, adopted variational autoencoder architecture to prune and enrich terms and decoder architecture to explain the genetic contributions of each term, with the latent space dimension equal to the number of terms. The model leverages a nonlinear encoder for flexibility and a linear decoder for interpretability, based on the publicly available model expiMap [31]. Considering the large scale and potential redundancy of perturbation data, the attention-like mechanism was implemented in latent space to focus on relevant perturbation terms for each cell (see Materials and Methods). Finally, based on the trained end-to-end model, we could predict the variation induced by term in the query data, as well as the measure affection of the genes in each term. The proposed framework takes advantage of large-scale perturbation datasets to explore and capture the gene expression variation induced by a diverse array of compounds at the single-cell level and quantify the contribution of affected genes in these variations.

To discover candidate drugs and their potential targets, we applied Shennong to our pan-cancer landscape and explored the response of the tumor cells to pharmacologic compounds. We first constructed the training set by randomly selecting ∼90% of cells (346 129 cells) in the pan-cancer landscape and HCL and the remaining cells (42 517 cells) as the prediction set to evaluate whether Shennong could correctly study the effect of single-cell perturbations. Both the training and prediction sets contained cells from tumor, adjacent, and healthy normal tissues, covering epithelial, endothelial, lymphoid, myeloid, and stromal cell lineages. Successful learning should extract distinct and common features of cells from different tissue sources and map those cells to various cell types while calculating the strong effect of terms. After training and prediction, we could successfully extract features from the training set and map them to the prediction set (Fig. 4b and Fig. S9b–d). Cells in the prediction set were divided into distinct clusters, and clusters from the same lineage tended to cluster together (Fig. 4b). The clustering and distribution of predicted cells were consistent with the cell type annotations in the single-lineage analyses of the pan-cancer landscape and HCL (Fig. 3a, f and Fig. S8a). These clusters originate primarily from a single tissue source or tissue type in epithelial cells, whereas clusters in other lineages span cells from different tissue sources and tissue types (Fig. S9b). Furthermore, we applied the trained framework to the entire number of cells in the pan-cancer landscape and HCL (388 646 cells) and found that predicted cells were greatly mapped onto the training cells that grouped into individual clusters and were close to cells from the same lineage (Fig. S9c, d). These indicated that the framework successfully learned the features of tumor and normal cells and was easy to transfer and accurately predict other data. Then we performed 10-fold cross-validation analysis, and the feature extraction and cell clustering were highly reproducible, indicating the robustness of the Shennong framework (Fig. S10).

Based on the influence term scores for each cell, which capture both the latent scores and directions, we identified significantly differential terms in each cell type using Bayes factors (Table S14). Using the top 10 significantly differential terms of each cell type, we could distinguish the different cell types in the single-lineage analyses (Fig. 4c and Fig. S11a, b). The term was a collection of significant features extracted from perturbation data that reflect the effects of the compound treatment on cells. Counting these top 10 terms, we found that some terms were observed multiple times (Fig. 4d, the compounds corresponding to the terms labeled), and small molecules corresponding to these terms have been subjected to extensive laboratory experiments or clinical trials in many cancer types. Notably, some of these compounds have even been approved by the United States Food and Drug Administration (FDA). For example, azacitidine, an inhibitor of DNA methylation, is approved for the treatment of myelodysplastic syndromes and acute myeloid leukemia, and has been in completed and ongoing clinical trials for glioma (NCT03666559), CRC, small-cell lung carcinomas, ovarian cancer, breast cancer, and pancreatic cancer (NCT03264404). Irinotecan is one of the most important cytotoxic anticancer drugs for the treatment of advanced cancers, in particular colon cancer and certain other solid tumors [32]. Tosedostat is an aminopeptidase inhibitor that has shown efficacy in clinical trials for the treatment of acute myeloid leukemia [33] and solid tumors [34]. Interestingly, top significantly differential terms were more recurrent in stromal and endothelial cells than in epithelial cells (Fig. 4e and Fig. S11c). CAFs (C1, C3, C4, and C7) had similar perturbation influences that differed from normal fibroblasts and smooth muscle cells (Fig. 4c), and similar results were also observed in endothelial cells (Fig. S11b). Through calculating the enrichment score of each term, we found that LJP008_SKL_24H: G19_DOWN and MOA001_U2OS_24H: P04_DOWN were the most recurrent significantly differential terms (Fig. 4e), corresponding to the significant features extracted from azacitidine and palbendazole. Both terms were enriched in CAFs instead of other stromal cells or cells of other lineages (Fig. 4f) and could separate CAFs from other cells (Fig. 4g). It suggests that azacitidine and palbendazole were sensitive to CAFs composed of cells from multiple tissues and may have the potential of pan-cancer therapies. Besides its FDA-approved indications, azacitidine has been in clinical trials for various cancer types. Parbendazole has been reported to be a repurposed drug candidate for the treatment of multiple cancers, including acute myeloid leukemia, pancreatic cancer, and head and neck squamous cell carcinoma [35,36], although it's FDA-approved for treating parasitic infections in animals. Furthermore, we found that the significantly differential terms CPC006_HT29_6H: D19_UP and CPC006_PC3_24H: J14_DOWN was recurrent in stromal cells and endothelial cells (Fig. 4e and Fig. S11c) and were particularly enriched in cancer-associated stromal cells and endothelial cells (Fig. 4h and Fig. S11d). Cancer-associated stromal cells and endothelial cells could be easily separated from normal stromal cells and endothelial cells or cells of other lineages by these two terms (Fig. 4i and Fig. S11e). The compound corresponding to CPC006_PC3_24H: J14_DOWN, erastin, is a ferroptosis inducer with potent anticancer activity and could induce iron-dependent cell death of cancer cells in solid cancers and blood cancers [37]. These results further indicated the effectiveness of the Shennong framework in discovering broad-spectrum anticancer drugs.

### Identifying anticancer drugs and potential targets with Shennong

**Figure 5.**
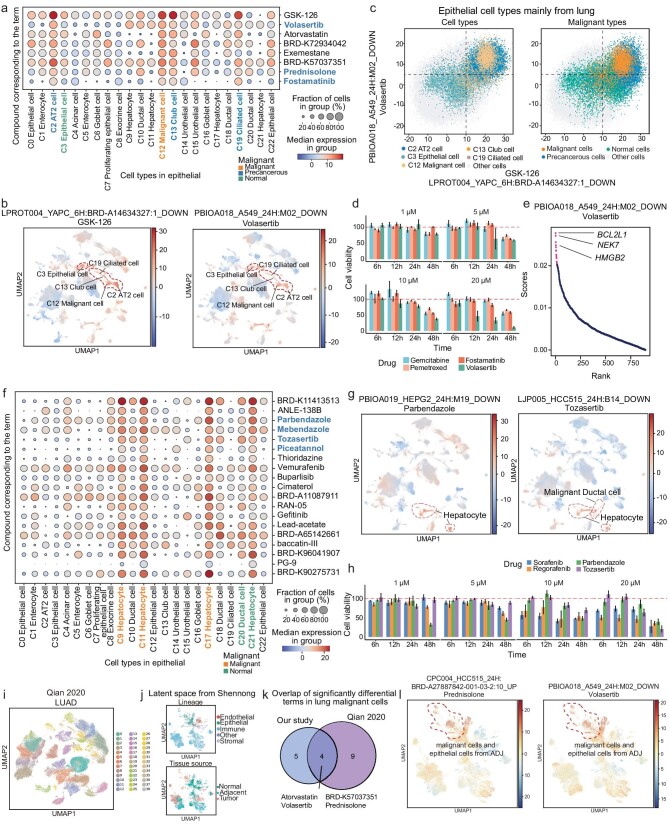
Identifying anticancer drugs and potential targets using Shennong. (a) Dot plot showing the influence term scores of terms across all epithelial cell types that are significantly different in epithelial cell types mainly from lung. Compounds corresponding to the terms and epithelial cell types mainly from lung are labeled. (b) UMAP representation of the influence term scores of all cells for terms LPROT004_YAPC_6H: BRD-A14634327: 1_DOWN (left) and PBIOA018_A549_24H: M02_DOWN (right), corresponding to the compounds GSK-126 and volasertib, respectively. (c) Visualization of cell types mainly from lung (left) and corresponding malignant types (right) in the terms mentioned in (b). Each dot shows the influence terms score of each cell. (d) Bar plot showing cell viability of A549 cell lines treated with the compounds gemcitabine, pemetrexed, fostamatinib, and volasertib across four dose regimens (1 μm, 5 μm, 10 μm and 20 μm) over time points of 6 hours (6 h), 12 h, 24 h and 48 h. Cell viability was assessed using a standard assay, with control cells receiving DMSO; data were presented as mean ± SEM for each treatment group at the indicated doses. (e) Dot plot showing the absolute weights of genes contribution to the term PBIOA018_A549_24H: M02_DOWN. (f) Dot plot showing the influence term scores of terms across all epithelial cell types that are significantly different in epithelial cell types mainly from liver. Compounds corresponding to the terms and epithelial cell types mainly from liver are labeled. (g) UMAP representation of the influence term scores of all cells for terms PBIOA019_HEPG2_24H: M19_DOWN (left) and LJP005_HCC515_24H: B14_DOWN (right) corresponding to the compounds parbendazole and tozasertib, respectively. (h) Bar plot showing cell viability of HepG2 cell lines treated with the compounds sorafenib, regorafenib, parbendazole and tozasertib across four dose regimens (1 μm, 5 μm, 10 μm and 20 μm) over time points of 6 h, 12 h, 24 h and 48 h. Cell viability was assessed using a standard assay, with control cells receiving DMSO; data were presented as mean ± SEM for each treatment group at the indicated doses. (i) UMAP visualization of cells from the LUAD dataset (112 176 cells), colored by cell clusters. (j) UMAP representation of cells in the LUAD dataset embedded in latent space extracted from the framework, colored by cell lineage (top) and tissue source (bottom). (k) Overlap of significantly different terms in lung malignant cells between pan-cancer landscape (C12) and the third-party LUAD dataset (clusters 15, 26, 29, and 35; only terms observed in at least two clusters were counted). (l) UMAP representation of influence term scores of all cells for terms CPC004_HCC515_24H: BRD-A27887842-001-03–2: 10_UP (left) and PBIOA018_A549_24H: M02_DOWN (right), corresponding to the compounds prednisolone and volasertib, respectively.

Unlike CAFs originating from different tissues, most malignant and precancerous cells were tissue-specific. The significantly differential terms were distinct in each epithelial cell type, but some were recurrent across cell types primarily originating from the same tissue (Fig. S12a). We found a series of terms significantly enriched in lung malignant cells and precancerous cells, but not in normal lung epithelial cells (Fig. 5a). The compounds corresponding to these terms include FDA-approval drugs, experimental drugs, and experimental compounds, most of which have been shown to inhibit tumor growth and tumor expansion, particularly in lung cancer. For example, the terms LPROT004_YAPC_6H: BRD-A14634327:1_DOWN and PBIOA018_A549_24H: M02_DOWN were enriched in malignant and precancerous cells, including AT2 cells (C2), club cells (C13), and ciliated cells (C19), corresponding to small molecular compounds GSK-126 and volasertib, respectively (Fig. 5b). Using these two terms, malignant and precancerous cells originating from the lung could be easily distinguished (Fig. 5c and Fig. S12b). In contrast, these terms had low influence scores in normal lung epithelial cells and in epithelial cells of other tissue, indicating the corresponding compounds were not sensitive to these cells.

GSK-126 (GSK2816126) is a potent and highly selective EZH2 inhibitor. Although a phase I clinical trial (NCT02082977) with 41 patients indicated insufficient clinical activity due to dosing limitations [38], preclinical studies have shown promising effects in lung cancer in both cell line and mouse experiments [39,40], suggesting potential efficacy in specific contexts. Volasertib is also an experimental drug that has demonstrated safety and antitumor activity in clinical trials [41]. For example, in a phase I trial (NCT00969761) involving 61 patients (14 with non-small cell lung cancer, NSCLC), volasertib combined with cisplatin or carboplatin showed an acceptable safety profile and resulted in stable disease in 17 patients, including 5 with NSCLC [42]. Additionally, a phase I dose-escalation study of volasertib combined with nintedanib reported a partial response in 1 of 5 patients with NSCLC [43]. Besides GSK-126 and volasertib, other drugs/compounds corresponding to the significantly differential terms include atorvastatin, exemestane, prednisolone, and fostamatinib (Fig. 5a). Atorvastatin is currently under investigation for its potential in cancer therapy [44], while exemestane has shown antiproliferative effects on lung cancer cells [45,46]. A phase Ib trial (NCT01664754) reported an objective response rate of 62.5% and the clinical benefit rate of 87.5% for exemestane in combination with chemotherapy in NSCLC patients, with the objective response rate significantly correlated with exemestane (*p* = 0.02) [47]. Fostamatinib has not been reported to have antitumor activity against lung cancer. To further investigate, we performed preliminary cell-based screenings on A549 cell lines treated with these compounds and chemotherapy agents (gemcitabine and pemetrexed) at four doses over 6 to 48 hours. Fostamatinib treatment resulted in decreased cell viability (Fig. 5d), confirming its anti-lung cancer activity. Notably, fostamatinib stood out due to its highly excellent low cell viability values on cell lines, which are under phase studies for the treatment of warm antibody autoimmune hemolytic anemia [48]. These results demonstrated the ability of our framework to screen anticancer drug candidates and identify new drug indications.

To investigate mechanisms of drug action, we investigated individual gene contributions within each term to elucidate potential targets of corresponding compounds and associated signaling pathways. For example, volasertib can cause downregulated *BCL2L1* expression, disrupt the interaction between PLK1 and *NEK7*, or displacement of *HMGB2* from mitotic chromosomes, thereby affecting mitotic arrest and apoptosis in cancer cells. These genes were significant contributors to term PBIOA018_A549_24H: M02_DOWN (Fig. 5e), which corresponds to the polo-like kinase 1 (PLK1) small molecule inhibitor drug volasertib. These indicated the efficiency and accuracy of the Shennong framework, as well as its ability to *in silico* screen anticancer drugs and explore action mechanisms. These results support future clinical trials to confirm antitumor efficacy with this combination therapy.

Next, we focused on terms that had great effects on malignant cells in liver and collected the top 10 significantly differential terms in malignant hepatocytes (Fig. 5f). The majority of the terms were significantly enriched in malignant hepatocytes but not in normal hepatocytes, such as PBIOA019_HEPG2_24H: M19_DOWN, CPC002_HCC515_24H: BRD-K77987382-001-08–2:10_DOWN, LJP005_HCC515_24H: B14_DOWN, and CPC017_HEPG2_6H: BRD-K91509126-001-04–6:10_DOWN (Fig. 5g and Fig. S12c). Compounds corresponding to these terms were parbendazole, mebendazole, tozasertib, and piceatannol. Malignant hepatocytes could be separated from other cells using these four terms (Fig. S12d, e), implicating the anticancer activity of these compounds in liver tumors.

Tozasertib (VX-680, MK-0457) is the first potent Aurora kinase inhibitor to undergo clinical trials and preclinical studies have shown that it can inhibit cell growth and increase apoptosis in solid tumors and leukemia [49,50]. And a phase I clinical trial (NCT00104351) with 21 patients indicated stable disease in a subset of patients, supporting its potential as a therapeutic agent [51]. Piceatannol is a phenolic compound, a hydroxylated analogue of resveratrol that has potent antioxidant activity and has chemopreventive and anticancer properties in cell line experiments [52]. Mebendazole and parbendazole are both FDA-approved drugs used to treat parasitic worm infections. Mebendazole has been extensively studied as a repurposed anticancer drug due to its interference with microtubule formations, which are essential for cell division [53,54]. Experiment and preclinical studies confirmed that mebendazole has antitumor activity against a variety of cancers and has entered the clinical stage. Like mebendazole, parbendazole is an oral anthelmintic that has shown preclinical efficacy against cancers [54], but parbendazole has not shown antitumor activity in liver cancer cells in previous studies. The cell-based screening on HepG2 cell lines confirmed the anti-liver cancer activity of parbendazole (Fig. 5h), comparable to standard therapies for advanced HCC (sorafenib and regorafenib), as well as the clinical trial drug tozasertib, which has demonstrated clear antitumor activity in clinical studies. Although tozasertib treatment has clear antitumor activity in phase I/II clinical trials, studies were terminated due to toxic adverse effects [55]. Based on the results of our framework, these compounds (drugs) corresponding to top significantly differential terms could be prioritized for repositioning as anticancer drug candidates, but challenges remained in large-scale clinical trials, such as the discovery of tozasertib.

To enhance the robustness of our framework, we compared the prediction results in our pan-cancer landscape (i.e. significant terms of malignant cells) with those in third-party datasets [56,57] (Fig. 5 i–l and Fig. S13a–d). The trained model successfully extracted distinct and common features of cells across different tissue sources in these datasets, and the latent space showed that clusters from the same lineage tended to cluster together after prediction by the Shennong framework (Fig. 5j and Fig. S13b). Notably, 30%∼45% of significantly differential terms in malignant cells from our pan-cancer landscape overlapped with those in corresponding third-party datasets (Fig. 5k and Fig. S13c). Only terms observed in at least two clusters were counted. These overlapping terms included the experimental drug volasertib and tozasertib (both in clinical trials), as well as the new candidates prednisolone and mebendazole (Fig. 5k and Fig. S13c). In summary, comparisons of the prediction results between our pan-cancer landscape and third-party datasets indicated that the framework was robust and exhibited great generalization capabilities.

### Evaluating tissue damaging effects of anticancer drugs using Shennong

Predicting potential side effects of anticancer drugs could improve the accuracy and efficiency of drug screening. When focusing on terms enriched in cancer-associated stromal cells and endothelial cells, we observed that CPC006_PC3_24H: J14_DOWN was also enriched in normal endothelial cells (C2 and C7) (Fig. 4h). This suggested the potential tissue damaging effects of the small molecular compound corresponding to the term. Erastin, corresponding to CPC006_PC3_24H: J14_DOWN, has demonstrated toxicity to healthy tissue in preclinical studies, particularly regarding ferroptosis induction [58]. This finding aligns with our model's predictions and raises potential concerns about its clinical application.

**Figure 6.**
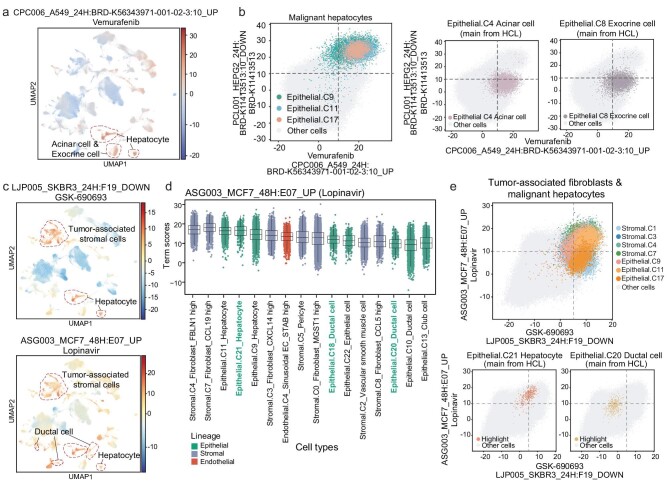
Identifying tissue damaging effects of anticancer drugs using Shennong. (a) UMAP representation of the influence term scores of all cells for the term CPC006_A549_24H: BRD-K56343971-001-02–3:10_UP, corresponding to the compound vemurafenib. (b) Visualization of malignant hepatocytes (left) and each epithelial cell type mainly from pancreas (right) in the context of the terms CPC006_A549_24H: BRD-K56343971-001-02–3:10_UP and PCL001_HEPG2_24H: BRD-K11413513:10_DOWN, corresponding to the compounds vemurafenib and BRD-K11413513. Each dot shows the influence terms score of each cell. (c) UMAP representation of the influence term scores of all cells for terms LJP005_SKBR3_24H: F19_DOWN (top) and ASG003_MCF7_48H: E07_UP (bottom), corresponding to compounds GSK-690693 and lopinavir, respectively. (d) Box plot showing influence score of the top 16 cell types in the term ASG003_MCF7_48H: E07_UP corresponding to the compound lopinavir. Epithelial cell types originating from HCL are colored green. (e) Visualization of tumor-associated fibroblasts or malignant hepatocytes (top) and each epithelial cell type mainly from liver (bottom) in the context of the terms LJP005_SKBR3_24H: F19_DOWN and ASG003_MCF7_48H: E07_UP. Each dot shows the influence terms score of each cell.

Additionally, we discovered that some terms were enriched not only in malignant hepatocytes but also in cell types mainly originating from HCL. These suggested that the small molecular compounds corresponding to those terms might also be sensitive to normal cells. For example, term CPC006_A549_24H: BRD-K56343971-001-02–3:10_UP had significantly high influence term scores in hepatocytes, acinar cells (C4), and exocrine cells (C8) (Fig. 6a, b). Unlike malignant hepatocytes, the acinar cells and exocrine cells originated from the normal pancreas tissues, suggesting that the related compound could have tissue damaging effects on normal pancreatic epithelial cells. The term corresponding compound vemurafenib is a BRAF inhibitor used as a targeted therapy for Erdheim-Chester disease and melanoma. There have been a few reported cases of pancreatitis associated with the use of vemurafenib (Zelboraf) [59]. A safety review initiated after identifying 18 cases of vemurafenib-associated pancreatitis during clinical trials underscores the importance of monitoring drug safety in patients. Consequently, the labeling for vemurafenib was updated in Europe and Canada to reflect these clinical implications.

In stromal and endothelial cells, LJP005_SKBR3_24H: F19_DOWN and ASG003_MCF7_48H: E07_UP were recurrent significantly differential terms (Fig. 4e and Fig. S11c). In addition to tumor-associated stromal cells and endothelial cells, these two terms had significantly highly influence term scores in malignant and precancerous epithelial cells, as well as normal fibroblasts and normal epithelial cells, such as fibroblast_MGST1 high (stromal, C0), hepatocytes (epithelial, C21), and ductal cells (epithelial, C18 and epithelial, C20) (Fig. 6c–e and Fig. S14a, b). This suggested that the compounds corresponding to these two terms had significant effects on normal liver epithelial and stromal cells.

Lopinavir corresponding to ASG003_MCF7_48H: E07_UP, is an antiretroviral drug of the protease inhibitor class that may cause liver injury. LJP005_SKBR3_24H: F19_DOWN was corresponding to GSK-690693, an ATP-competitive pan-Akt inhibitor. In preclinical studies, GSK-690693 was shown to inhibit the proliferation of multiple cancers, including various hematologic neoplasia [60], but clinical trials have been withdrawn or terminated (NCT00666081 and NCT00493818), most likely due to modest antitumor activity [61] and side-effects associated with transient hyperglycemia [62]. There is no doubt that the term corresponding compound was sensitive to normal hepatocytes, acinar cells, and exocrine cells, which play important roles in the insulin signaling pathway. These findings underscore the effectiveness of the Shennong framework in the prediction of tissue damaging effects.

## DISCUSSION

Pan-cancer landscape is an essential resource for advancing our understanding of cancer biology and improving diagnosis and treatment. In this study, we used Microwell-seq to profile the pan-cancer single-cell landscape covering 303 351 cells, which contained the highest morbidity and mortality tumor types in China. This extensive dataset, combined with normal healthy tissue data from our previous work [7], allowed for a robust identification of malignant and precancerous cells, enhancing our confidence in the defined cellular states.

Our findings indicated that precancerous cells, originating not only from ADJ tissues but also from CA tissues, reflect intermediate states from normal to tumor. These precancerous cells exhibit molecular alterations associated with nearby tumors, suggesting they play a significant role in tumorigenesis. The shared pathways and metabolic patterns between precancerous and malignant cells underscore the necessity of differentiating these cell types, as they possess unique characteristics distinct from normal epithelial cells, which were hidden in bulk RNA sequencing.

Additionally, we comprehensively characterized cancer-associated fibroblasts and endothelial cells through pan-cancer analyses. The expression analyses and regulatory patterns revealed similarities between stromal or endothelial cells from CA and ADJ tissues, confirming the distinct functionalities of cells from adjacent and normal tissues.

To enhance accessibility and usability, we developed an interactive portal (http://bis.zju.edu.cn/shennong/landscape.html) for visualization and querying of our integrative dataset.

Traditional methods for drug screening or drug discovery are time-consuming and costly. Artificial intelligence and machine learning methods accelerate the process of drug discovery and development. The Shennong framework, designed for *in silico* screening of anticancer drugs at the single-cell level, exemplifies this approach. This robust and explainable framework enables us to predict cellular responses to pharmacologic compounds, screen drug candidates, evaluate drug candidates’ tissue damaging effects, and explore their action mechanisms.

Through our analysis, we identified FDA-approved drugs with novel indications for cancer treatment, such as azacitidine and irinotecan, showing the framework's potential for drug repurposing. Moreover, our predictions of tissue damaging effects, which were consistent with reported clinical injuries, demonstrated the framework's capacity for fine-grained analysis of drug effects, particularly in healthy tissues. For example, our results indicated that vemurafenib may adversely affect normal pancreatic epithelial cells, highlighting the importance of considering tissue damaging effects in drug development.

Despite the promising results, we acknowledge certain limitations inherent in our study. A critical concern is the reliance on perturbation data. While CMap provides valuable information, it primarily contains molecules with experimentally assessed transcriptional profiles. The differences in data collection platforms may introduce batch effects, and the perturbation data, derived from bulk RNA-seq, do not capture the influence on specific cell types, which could affect the generalizability of our findings. Our framework focuses on significant features that change with perturbations, which may limit its applicability if the perturbation datasets are not sufficiently comprehensive. Future enhancements to the framework could benefit from larger and more diverse perturbation datasets, including ligands and siRNAs, to refine predictions and deepen our understanding of cellular responses at the single-cell level. Additionally, drug treatment data from mouse models and patients could provide valuable perturbations for future studies. Moreover, many common side effects manifest as systemic reactions throughout the body, including fatigue, hair loss, allergic reactions, nausea, and anemia. In our analysis, the tissue damaging effects predominantly focus on the potential cytotoxic effects on specific cell types. This framework might only be partially adequate for predicting side effects, as it does not fully guarantee or anticipate all potential adverse reactions.

In this context, we note that although cellular experiments, comparisons with third-party datasets, and linked recent clinical data support the robustness of our predictions, experimental validation is still needed. Follow-up studies, including *in vitro* or animal model experiments, are essential to confirm the biological effects, safety, and efficacy of these predicted components.

In conclusion, our study made some substantial progress in cancer research and drug development through the Shennong framework, which provides single-cell predictions to identify specific target cells and genes, as well as novel indications, potential drug resistance mechanisms, and tissue damaging effects. This framework is particularly beneficial for personalized treatment, especially for patients with rare cancer subtypes, as it enhances efficiency and cost-effectiveness in drug discovery by saving time and resources. The use of perturbation data allows the framework to have high accuracy in drug repurposing. Although some predicted compounds were discontinued in clinical trials due to antitumor activity, highlighting the gap between clinical practice and laboratory research, we successfully predicted several FDA-approved drugs currently in trials for other cancer types. This demonstrates strong application potential and predictive accuracy, which can be accessed on our website, offering new insights and directions for targeted therapies and personalized medicine.

## MATERIALS AND METHODS

### Patients and sample collection

All patients gave their written informed consent for scientific evaluations. The study was approved by the Ethics Committee of the First Affiliated Hospital and the Second Affiliated Hospital, Zhejiang University School of Medicine (IIT20210078B). The cancer and adjacent paracancerous tissue samples required for the experiment were obtained from the patients after surgery, and stored in DMEM (Dulbecco's modified eagle’s medium, ThermoFisher) at 4°C, and the scRNA-seq was performed within 2 hours. Detailed clinical information for these patients is provided in Table S1.

### scRNA sequencing and data processing

We used the Microwell-seq process to obtain single-cell RNA data for each sample. The Microwell-seq process included cell collection and lysis, reverse transcription, exonuclease I treatment, second-strand synthesis, cDNA amplification and transposase fragmentation, and selective PCR to generate barcoded single-cell libraries. The samples were subjected to sequencing on the MGI DNBSEQ-T7. We also replaced the official R1 sequencing primers with our customized R1 sequencing primers A and B (listed in Table S4) to ensure the completion of the sequencing. Raw Microwell-seq data were processed following the protocols in our previously published work [7,63]. Reads were aligned to the *Homo sapiens* GRCh38 genome. After filtering, dimension reduction, clustering, and differential gene expression analysis was performed on the processed DGE data using Seurat and Scanpy. Detailed methods are described in Supplementary Materials and Methods.

### Malignant cell identification

Malignant cells were identified simultaneously using three methods. First, the DGE data of each patient were merged and clustered using the Seurat pipeline, cell types were classified into five lineages, including epithelial, endothelial, stromal, lymphoid, and myeloid. For epithelial cell types, the expression patterns of each cluster were examined to distinguish potential malignancy. Genes that were overexpressed in malignant relative to normal tissue for each cancer type were examined. Second, RNA-based copy-number variation inference was performed on all epithelial cells for each patient in the inferCNV package, using epithelial cells of corresponding normal tissues of the cancer type in HCL as a reference. Third, dimensionality reduction was performed on the potential malignant cells from different cancer types, which should form separate clusters. Detailed methods are described in Supplementary Materials and Methods.

### Shennong analytical workflow

The Shennong framework consists of three main stages (Fig. 4a). In the first stage, the merged scRNA-seq count matrix was integrated with the preprocessed perturbation data to obtain perturbation change features. The perturbation data was saved in gmt file, each line in this file corresponds to a term, which represents a unique and specific gene set associated with particular compounds and experimental conditions in our study.

In the second stage, Shennong established a cellular perturbation predictive model to capture cellular responses to pharmacological perturbations at the single-cell level. The variational autoencoder architecture was adopted to incorporate individual cells from different conditions and ensure full capture of term variability, based on publicly available model expiMap [31]. The model contained 4 hidden layers for the encoder network and the same layers in reversed order for the decoder network, with non-linear encoder for flexibility and masked linear decoder for interoperability. In the bottleneck network, the latent space dimension was equal to term numbers. The model was trained on reference scRNA-seq data and perturbation data.

In the third stage, the influence induced by each term on each cell was measured based on the trained end-to-end model. The absolute values of decoder weights for genes in each term were extracted and ranked to measure the genetic contributions of each term.

Detailed methods are described in Supplementary Materials and Methods.

### Application of Shennong to the pan-cancer landscape

A total of 86 cell clusters from 388 646 cells across 6 tissues were collected. A total of 346 129 cells were selected for the training set and the rest for the prediction set. The training set was integrated with preprocessed perturbation data to generate a perturbation binary matrix, which was then fed into deep learning. A series of pre-training to optimize the hyperparameters of the model on the training set, alpha_kl = 0.005, alpha = 0.95, and hidden_layer_sizes = 512 were used.

In prediction, features from the prediction set were extracted and mapped well to the training set (Fig. 4b and Fig. S9b). Then entire amount cells of pan-cancer landscape and HCL were fed into the prediction model, and the latent space visualization showed great integration (Fig. S9c, d). The enrichment test was performed using Bayes Factors to identify significantly differential terms across. A term was considered significantly different if its absolute log-Bayes factor was >2.3, which is referred to as the enrichment score. The latent scores of a term in all cells were visualized in UMAP, as well as the latent variables of two terms in all cells. The gene contributions of each term were extracted from the decoder and sorted by their absolute weight, visualized in the dot plot.

Detailed methods are described in Supplementary Materials and Methods. The training and prediction results can be obtained and queried on our website (http://bis.zju.edu.cn/shennong/index.html).

## Supplementary Material

nwae451_Supplemental_File

## Data Availability

The raw sequence data reported in this paper have been deposited in the Genome Sequence Archive in National Genomics Data Center, China National Center for Bioinformation/Beijing Institute of Genomics, Chinese Academy of Sciences (GSA-Human: HRA006591) that are publicly accessible at https://ngdc.cncb.ac.cn/gsa-human. The data deposited and made public are compliant with the regulations of the Ministry of Science and Technology of China. Processed count matrices and cell annotations are available at figshare: https://figshare.com/s/ac34f719115943d1d46c. Single patient scRNA-seq data for LUAD and PDAC were obtained from the Gene Expression Omnibus (GEO) database, with accession numbers GSE131907 and GSE155698, respectively. All cell type labels and metadata were obtained from original publications. For the third-party comparison scRNA-seq data, LUAD data was obtained from the ArrayExpress database (accession number E-MTAB-6149) while HCC data was obtained from GEO (accession number GSE1496140). The metadata was obtained from original publications, and the cell type labels were reclustered and annotated according to those publications.

## CODE AVAILABILITY

The source code for reproducing our analysis and training the Shennong framework is available at figshare (https://figshare.com/s/ac34f719115943d1d46c) and GitHub (https://github.com/PeijingZhang/Shennong).

## Notes

***Conflict of intereststatement.*** None declared.

## References

[1] McGranahan N, Swanton C. Biological and therapeutic impact of intratumor heterogeneity in cancer evolution. Cancer Cell 2015; 27: 15–26. 10.1016/j.ccell.2014.12.001 25584892

[2] Barkley D, Moncada R, Pour M et al. Cancer cell states recur across tumor types and form specific interactions with the tumor microenvironment. Nat Genet 2022; 54: 1192–201. 10.1038/s41588-022-01141-9 35931863 PMC9886402

[3] Priestley P, Baber J, Lolkema MP et al. Pan-cancer whole-genome analyses of metastatic solid tumours. Nature 2019; 575: 210–6. 10.1038/s41586-019-1689-y 31645765 PMC6872491

[4] Kinker GS, Greenwald AC, Tal R et al. Pan-cancer single-cell RNA-seq identifies recurring programs of cellular heterogeneity. Nat Genet 2020; 52: 1208–18. 10.1038/s41588-020-00726-6 33128048 PMC8135089

[5] Zheng L, Qin S, Si W et al. Pan-cancer single-cell landscape of tumor-infiltrating T cells. Science 2021; 374: abe6474. 10.1126/science.abe6474 34914499

[6] Han X, Wang R, Zhou Y et al. Mapping the mouse cell atlas by Microwell-Seq. Cell 2018; 173: 1307. 10.1016/j.cell.2018.05.012 29775597

[7] Han X, Zhou Z, Fei L et al. Construction of a human cell landscape at single-cell level. Nature 2020; 581: 303–9. 10.1038/s41586-020-2157-4 32214235

[8] Vamathevan J, Clark D, Czodrowski P et al. Applications of machine learning in drug discovery and development. Nat Rev Drug Discov 2019; 18: 463–77. 10.1038/s41573-019-0024-5 30976107 PMC6552674

[9] Tran KA, Kondrashova O, Bradley A et al. Deep learning in cancer diagnosis, prognosis and treatment selection. Genome Med 2021; 13: 152. 10.1186/s13073-021-00968-x 34579788 PMC8477474

[10] You Y, Lai X, Pan Y et al. Artificial intelligence in cancer target identification and drug discovery. Signal Transduct Target Ther 2022; 7: 156. 10.1038/s41392-022-00994-0 35538061 PMC9090746

[11] Subramanian A, Narayan R, Corsello SM et al. A next generation connectivity map: L1000 platform and the first 1,000,000 profiles. Cell 2017; 171: 1437–52 e1417. 10.1016/j.cell.2017.10.049 29195078 PMC5990023

[12] Li C, Shao X, Zhang S et al. scRank infers drug-responsive cell types from untreated scRNA-seq data using a target-perturbed gene regulatory network. Cell Rep Med 2024; 5: 101568. 10.1016/j.xcrm.2024.101568 38754419 PMC11228399

[13] Gavish A, Tyler M, Greenwald AC et al. Hallmarks of transcriptional intratumour heterogeneity across a thousand tumours. Nature 2023; 618: 598–606. 10.1038/s41586-023-06130-4 37258682

[14] Huang XY, Ke AW, Shi GM et al. Overexpression of CD151 as an adverse marker for intrahepatic cholangiocarcinoma patients. Cancer 2010; 116: 5440–51. 10.1002/cncr.25485 20715158

[15] Kim N, Kim HK, Lee K et al. Single-cell RNA sequencing demonstrates the molecular and cellular reprogramming of metastatic lung adenocarcinoma. Nat Commun 2020; 11: 2285. 10.1038/s41467-020-16164-1 32385277 PMC7210975

[16] Steele NG, Carpenter ES, Kemp SB et al. Multimodal mapping of the tumor and peripheral blood immune landscape in Human pancreatic cancer. Nat Cancer 2020; 1: 1097–112. 10.1038/s43018-020-00121-4 34296197 PMC8294470

[17] Curtius K, Wright NA, Graham TA. An evolutionary perspective on field cancerization. Nat Rev Cancer 2018; 18: 19–32. 10.1038/nrc.2017.102 29217838

[18] Xia L, Oyang L, Lin J et al. The cancer metabolic reprogramming and immune response. Mol Cancer 2021; 20: 28. 10.1186/s12943-021-01316-8 33546704 PMC7863491

[19] Arner EN, Rathmell JC. Metabolic programming and immune suppression in the tumor microenvironment. Cancer Cell 2023; 41: 421–33. 10.1016/j.ccell.2023.01.009 36801000 PMC10023409

[20] Aran D, Camarda R, Odegaard J et al. Comprehensive analysis of normal adjacent to tumor transcriptomes. Nat Commun 2017; 8: 1077. 10.1038/s41467-017-01027-z 29057876 PMC5651823

[21] Li L, Ameri AH, Wang S et al. EGR1 regulates angiogenic and osteoclastogenic factors in prostate cancer and promotes metastasis. Oncogene 2019; 38: 6241–55. 10.1038/s41388-019-0873-8 31312026 PMC6715537

[22] Itahashi K, Irie T, Yuda J et al. BATF epigenetically and transcriptionally controls the activation program of regulatory T cells in human tumors. Sci Immunol 2022; 7: eabk0957. 10.1126/sciimmunol.abk0957 36206353

[23] Ma C, Yang C, Peng A et al. Pan-cancer spatially resolved single-cell analysis reveals the crosstalk between cancer-associated fibroblasts and tumor microenvironment. Mol Cancer 2023; 22: 170. 10.1186/s12943-023-01876-x 37833788 PMC10571470

[24] Augsten M, Hagglof C, Olsson E et al. CXCL14 is an autocrine growth factor for fibroblasts and acts as a multi-modal stimulator of prostate tumor growth. Proc Natl Acad Sci USA 2009; 106: 3414–9. 10.1073/pnas.0813144106 19218429 PMC2651265

[25] Friedman G, Levi-Galibov O, David E et al. Cancer-associated fibroblast compositions change with breast cancer progression linking the ratio of S100A4(+) and PDPN(+) CAFs to clinical outcome. Nat Cancer 2020; 1: 692–708. 10.1038/s43018-020-0082-y 35122040 PMC7617059

[26] Cords L, Tietscher S, Anzeneder T et al. Cancer-associated fibroblast classification in single-cell and spatial proteomics data. Nat Commun 2023; 14: 4294. 10.1038/s41467-023-39762-1 37463917 PMC10354071

[27] Luo H, Xia X, Huang LB et al. Pan-cancer single-cell analysis reveals the heterogeneity and plasticity of cancer-associated fibroblasts in the tumor microenvironment. Nat Commun 2022; 13: 6619. 10.1038/s41467-022-34395-2 36333338 PMC9636408

[28] Geldhof V, de Rooij L, Sokol L et al. Single cell atlas identifies lipid-processing and immunomodulatory endothelial cells in healthy and malignant breast. Nat Commun 2022; 13: 5511. 10.1038/s41467-022-33052-y 36127427 PMC9489707

[29] Poisson J, Lemoinne S, Boulanger C et al. Liver sinusoidal endothelial cells: physiology and role in liver diseases. J Hepatol 2017; 66: 212–27. 10.1016/j.jhep.2016.07.009 27423426

[30] Solimando AG, Summa S, Vacca A et al. Cancer-associated angiogenesis: the endothelial cell as a checkpoint for immunological patrolling. Cancers (Basel) 2020; 12: 3380. 10.3390/cancers12113380 33203154 PMC7696032

[31] Lotfollahi M, Rybakov S, Hrovatin K et al. Biologically informed deep learning to query gene programs in single-cell atlases. Nat Cell Biol 2023; 25: 337–50. 36732632 10.1038/s41556-022-01072-x PMC9928587

[32] Bailly C . Irinotecan: 25 years of cancer treatment. Pharmacol Res 2019; 148: 104398. 10.1016/j.phrs.2019.104398 31415916

[33] Lowenberg B, Morgan G, Ossenkoppele GJ et al. Phase I/II clinical study of Tosedostat, an inhibitor of aminopeptidases, in patients with acute myeloid leukemia and myelodysplasia. J Clin Oncol 2010; 28: 4333–8. 10.1200/JCO.2009.27.6295 20733120

[34] Reid AH, Protheroe A, Attard G et al. A first-in-man phase i and pharmacokinetic study on CHR-2797 (Tosedostat), an inhibitor of M1 aminopeptidases, in patients with advanced solid tumors. Clin Cancer Res 2009; 15: 4978–85. 10.1158/1078-0432.CCR-09-0306 19638462

[35] Matsuo H, Inagami A, Ito Y et al. Parbendazole as a promising drug for inducing differentiation of acute myeloid leukemia cells with various subtypes. Commun Biol 2024; 7: 123. 10.1038/s42003-024-05811-8 38267545 PMC10808455

[36] Liang D, Yu C, Ma Z et al. Identification of anthelmintic parbendazole as a therapeutic molecule for HNSCC through connectivity map-based drug repositioning. Acta Pharm Sin B 2022; 12: 2429–42. 10.1016/j.apsb.2021.12.005 35646536 PMC9136614

[37] Jiang X, Stockwell BR, Conrad M. Ferroptosis: mechanisms, biology and role in disease. Nat Rev Mol Cell Biol 2021; 22: 266–82. 10.1038/s41580-020-00324-8 33495651 PMC8142022

[38] Yap TA, Winter JN, Giulino-Roth L et al. Phase I study of the novel enhancer of Zeste Homolog 2 (EZH2) inhibitor GSK2816126 in patients with advanced hematologic and solid tumors. Clin Cancer Res 2019; 25: 7331–9. 10.1158/1078-0432.CCR-18-4121 31471312 PMC7377921

[39] McCabe MT, Ott HM, Ganji G et al. EZH2 inhibition as a therapeutic strategy for lymphoma with EZH2-activating mutations. Nature 2012; 492: 108–12. 10.1038/nature11606 23051747

[40] Duan R, Du W, Guo W. EZH2: a novel target for cancer treatment. J Hematol Oncol 2020; 13: 104. 10.1186/s13045-020-00937-8 32723346 PMC7385862

[41] Gjertsen BT, Schoffski P. Discovery and development of the Polo-like kinase inhibitor volasertib in cancer therapy. Leukemia 2015; 29: 11–9. 10.1038/leu.2014.222 25027517 PMC4335352

[42] Awada A, Dumez H, Aftimos PG et al. Phase I trial of volasertib, a Polo-like kinase inhibitor, plus platinum agents in solid tumors: safety, pharmacokinetics and activity. Invest New Drugs 2015; 33: 611–20. 10.1007/s10637-015-0223-9 25794535 PMC4435638

[43] de Braud F, Cascinu S, Spitaleri G et al. A phase I, dose-escalation study of volasertib combined with nintedanib in advanced solid tumors. Ann Oncol 2015; 26: 2341–6. 10.1093/annonc/mdv354 26395347

[44] Amin F, Fathi F, Reiner Z et al. The role of statins in lung cancer. Arch Med Sci 2022; 18: 141–52. 35154535 10.5114/aoms/123225 PMC8826694

[45] Koutras A, Giannopoulou E, Kritikou I et al. Antiproliferative effect of exemestane in lung cancer cells. Mol Cancer 2009; 8: 109. 10.1186/1476-4598-8-109 19930708 PMC2789046

[46] Collins IM, Nicholson SA, O'Byrne KJ. A lung cancer responding to hormonal therapy. J Thorac Oncol 2010; 5: 749–50. 10.1097/JTO.0b013e3181d1271d 20421769

[47] Young PA, Marquez-Garban DC, Noor ZS et al. Investigation of combination treatment with an aromatase inhibitor exemestane and carboplatin-based therapy for postmenopausal women with advanced NSCLC. JTO Clin Res Rep 2021; 2: 100150. 34590007 10.1016/j.jtocrr.2021.100150 PMC8474426

[48] Kuter DJ, Piatek C, Roth A et al. Fostamatinib for warm antibody autoimmune hemolytic anemia: phase 3, randomized, double-blind, placebo-controlled, global study (FORWARD). Am J Hematol 2024; 99: 79–87. 10.1002/ajh.27144 37929318

[49] Martens S, Goossens V, Devisscher L et al. RIPK1-dependent cell death: a novel target of the Aurora kinase inhibitor Tozasertib (VX-680). Cell Death Dis 2018; 9: 211. 10.1038/s41419-017-0245-7 29434255 PMC5833749

[50] Beinhoff P, Sabharwal L, Udhane V et al. Second-generation Jak2 inhibitors for advanced prostate cancer: are we ready for clinical development? Cancers (Basel) 2021; 13: 5204. 10.3390/cancers13205204 34680353 PMC8533841

[51] Traynor AM, Hewitt M, Liu G et al. Phase I dose escalation study of MK-0457, a novel Aurora kinase inhibitor, in adult patients with advanced solid tumors. Cancer Chemother Pharmacol 2011; 67: 305–14. 10.1007/s00280-010-1318-9 20386909 PMC3050703

[52] Banik K, Ranaware AM, Harsha C et al. Piceatannol: a natural stilbene for the prevention and treatment of cancer. Pharmacol Res 2020; 153: 104635. 10.1016/j.phrs.2020.104635 31926274

[53] Hegazy SK, El-Azab GA, Zakaria F et al. Mebendazole; from an anti-parasitic drug to a promising candidate for drug repurposing in colorectal cancer. Life Sci 2022; 299: 120536. 10.1016/j.lfs.2022.120536 35385794

[54] Joe NS, Godet I, Milki N et al. Mebendazole prevents distant organ metastases in part by decreasing ITGβ4 expression and cancer stemness. Breast Cancer Res 2022; 24: 98. 10.1186/s13058-022-01591-3 36578038 PMC9798635

[55] Cheung CH, Sarvagalla S, Lee JY et al. Aurora kinase inhibitor patents and agents in clinical testing: an update (2011–2013). Expert Opin Ther Pat 2014; 24: 1021–38. 10.1517/13543776.2014.931374 24965505

[56] Qian J, Olbrecht S, Boeckx B et al. A pan-cancer blueprint of the heterogeneous tumor microenvironment revealed by single-cell profiling. Cell Res 2020; 30: 745–62. 10.1038/s41422-020-0355-0 32561858 PMC7608385

[57] Lu Y, Yang A, Quan C et al. A single-cell atlas of the multicellular ecosystem of primary and metastatic hepatocellular carcinoma. Nat Commun 2022; 13: 4594. 10.1038/s41467-022-32283-3 35933472 PMC9357016

[58] Zhao J, Xu B, Xiong Q et al. Erastin‑induced ferroptosis causes physiological and pathological changes in healthy tissues of mice. Mol Med Rep 2021; 24: 713. 10.3892/mmr.2021.12352 34396451 PMC8383038

[59] Chung SY, Shen JG, Ghiuzeli CM. Vemurafenib-induced pancreatitis in a patient with recurrent hairy cell leukaemia. BMJ Case Rep 2020; 13: e236073. 10.1136/bcr-2020-236073 PMC7490934 32928833

[60] Levy DS, Kahana JA, Kumar R. AKT inhibitor, GSK690693, induces growth inhibition and apoptosis in acute lymphoblastic leukemia cell lines. Blood 2009; 113: 1723–9. 10.1182/blood-2008-02-137737 19064730

[61] Carol H, Morton CL, Gorlick R et al. Initial testing (stage 1) of the Akt inhibitor GSK690693 by the pediatric preclinical testing program. Pediatr Blood Cancer 2010; 55: 1329–37. 10.1002/pbc.22710 20740623 PMC2965797

[62] Crouthamel MC, Kahana JA, Korenchuk S et al. Mechanism and management of AKT inhibitor-induced hyperglycemia. Clin Cancer Res 2009; 15: 217–25. 10.1158/1078-0432.CCR-08-1253 19118049

[63] Wang R, Zhang P, Wang J et al. Construction of a cross-species cell landscape at single-cell level. Nucleic Acids Res 2023; 51: 501–16. 10.1093/nar/gkac633 35929025 PMC9881150

