# Supplementary material for: A deep learning framework for in silico screening of anticancer drugs at the single-cell level
Source: Natl Sci Rev. 2024 Dec 10;12(2):nwae451. doi: 10.1093/nsr/nwae451 (PMC11771446; doi:10.1093/nsr/nwae451)
Supplement: nwae451_Supplemental_File [file nwae451_supplemental_file.zip › Supplementary files/Supplementary files/Shennong-manuscript-s-20241126.docx]

## Supplementary Materials and Methods

### Sample preparation

The samples were quickly transferred into cold DPBS, and then minced into ∼1 mm pieces on ice with scissors. Tissue pieces were transferred to a 15 mL centrifuge tube, rinsed twice with cold DPBS and then resuspended with 5 mL of dissociation enzymes. Samples were treated with different enzymes for different durations. During the dissociation, tissue pieces were pipetted up and down gently for several times until there was no visible tissue fragment. The methods for single cell isolation from different tissues are listed in Supplementary Table 2. If the pellet is particularly red, the cells were suspended with 300 µL of PBS and 900µL of red blood cell lysis buffer (TIANGEN) with 5 min incubation on ice and the lysis reaction was quenched by adding 14 mL of DPBS with 2 mM EDTA. Otherwise, skip this step. Dissociated cells were centrifuged at 300 × g for 5 min at 4°C and suspended in 5 mL of DPBS with 2 mM EDTA. After passing through 40 μm strainers (Biologix), cells were washed twice, centrifuged at 300 × g for 5 min at 4°C, and re-suspended in cold DPBS with 2 mM EDTA at a density of 2 × 10^5^ cells/mL.

Bone marrow cells were isolated from femur and tibia bones. Muscles were removed from bones with Delicate Task Wipers. Both ends of the bones were carefully trimmed to expose the interior marrow shaft. We then used DPBS with 2 mM EDTA to flush the marrow using 1 mL syringe (with 26G needle). Marrow was collected into a 15 mL centrifuge tube, centrifuged at 300 × g for 5 min at 4°C, and re-suspended with DPBS. The supernatant was then collected to the same 15 mL centrifuge tube. After that, red blood cells are removed with red blood cell lysis buffer. red blood cell lysis buffer. Finally, the cells were collected and re-suspended with 3 mL of DPBS. After passing through 40 μm strainers, cells were washed twice, counted with a hemocytometer, and diluted to 2 × 10^5^ cells/mL in DPBS with 2 mM EDTA.

### Cell collection, lysis and reverse transcription

Single-cell complementary DNA libraries were prepared using the Microwell-seq. Briefly, cells were loaded on the microwell plate and extra cells were washed away gently using ice-cold PBS. Then bead suspension (sequences listed in Supplementary Table 3) was loaded on the plate and extra beads were washed away on a magnet. The plate was covered using cold lysis buffer (0.1 M Tris-HCl, pH 7.5, 0.5 M LiCl, 1% SDS, 10 mM EDTA and 5 mM dithiothreitol (DTT)) and incubated on ice for 15 min. Then, beads were collected and washed using 6 × saline sodium citrate and 50 mM Tris-HCl, pH 8.0. After washing, beads were resuspended in reverse transcription (RT) mix and incubated at 42°C for 90 min. The RT mix contained 200 U SuperScript II reverse transcriptase, 1× Superscript II first-strand buffer (Takara), 40 U Murine RNase inhibitor (Vazyme), 1 M betaine (Sigma), 6 mM MgCl_2_ (Ambion), 2.5 mM dithiothreitol, 1 mM deoxynucleoside triphosphate, and 1 µM TSO LNA primer (the sequences information is included in Supplementary Table 3).

### Exonuclease I treatment

After RT, beads were washed in 200 μl TE-SDS (1× TE + 0.5% SDS), 200 μl TE–TW (10 mM Tris-HCl, pH 8.0, 1 mM EDTA, 0.01% Tween20) and 200 μl 10 mM Tris-HCl, pH 8.0. Beads were resuspended in exonuclease I mix and incubated at 37°C for 30 min. The exonuclease I mix containing 1× exonuclease I buffer and 50 U exonuclease I (NEB). Then, beads were washed in TE–SDS, TE–TW and 10 mM Tris-HCl, pH 8.0.

### Second-strand synthesis

The beads were resuspended and incubated in 200 µL 0.1 M NaOH at room temperature for 30 secs and washed twice with 1mL TE-TW and once with 200 µL of 10 mM Tris-HCl (pH 8.0). The beads were resuspended in 20 µL dn-TSO oligo mix containing 5mM dn-TSO oligo, 10 mM Tris-HCl (pH 8.0) and 3mM MgCl2. The beads were then incubated in 95°C water bath for 30 seconds followed by mixing on a rotary mixer (10 rpm) at room temperature for 10 min to promote the binding of dn-TSO oligos (the sequences information is included in Supplementary Table 3). After that, the beads were washed once with 10 mM Tris-HCl (pH 8.0), and then combined with a 50ul master mix consisting of 1x RT buffer (Thermo), 12% PEG8000 solution, 1mM dNTPs, and 5 µL Klenow Exo- (Vazyme). Second-strand synthesis was carried out by incubating the beads for 1 hour at 37°C on a rotary mixer (10 rpm). The beads were then washed twice with 200 µL TE-TW and once with 10 mM Tris-HCl (pH 8.0).

### cDNA amplification

Beads were resuspended in PCR mix with TSO (template switch oligo) primer to amplify the cDNA. After PCR, beads were removed and cDNA products were purified using 0.9 × VAHTS DNA Clean Beads (Vazyme, catalog no. N411-01). A more detailed version of the Microwell-seq protocol is available in Han *et al* [1].

### Transposase fragmentation and selective PCR

Then, the purified cDNA libraries were fragmented using a customized transposase that carries two identical insertion sequences. The customized transposase was included in the TruePrep Homo-N7 DNA Library Prep Kit for MGI (Vazyme, catalog no. L-N7E461L0). The fragmentation reaction was performed according to the instructions provided by the manufacturer. We used customized MGI P5 primer (listed in Supplementary Table 4) and MGI P7 primers (listed in Supplementary Table 4) to specifically amplify fragments that contain the 3’-ends of transcripts. Other fragments will form self-loops, impeding their binding to PCR primers. To eliminate primer dimers and large fragments, 0.55×+ 0.15×VAHTS DNA Clean beads were then used to purify the cDNA library. The size distribution of the products was analyzed on an Agilent 2100 bioanalyzer, and a peak in the range 400–700 bp was observed. Finally, the samples were subjected to sequencing on the MGI DNBSEQ-T7. For MGI sequencing, we applied the protocol provided by the VAHTS Circularization Kit for MGI (Vazyme, catalog no. NM201-01) to obtain single-stranded circular cDNA available for DNB (DNA Nanoball) generation. We also replaced the official R1 sequencing primers with our customized R1 sequencing primers A and B (listed in Supplementary Table 4) to ensure the completion of the sequencing.

### Evaluation of cell viability

Evaluating cellular viability via the ATP bioluminescence assay (CellCounting-Lite® 2.0 Luminescent Cell Viability Assay kit, Vazyme, DD1101). Cells are resuspended in DMEM at 4,000 counts per well and seeded onto a 384 well plate (50 μL/well), incubated overnight. Drugs at final concentrations of either 1 μM, 5 μM, 10 μM or 20 μM are added to indicated groups and further incubated for 6, 12, 24 or 48 hours. Post treatment, 20 uL of the ATP detection reagent is added to each well, shaking at 500 rpm for 2-3 minutes at room temperature prior to assessment via the Thermo Scientific™ Varioskan™ LUX multimode microplate reader under 100 ms reading time conditions. The percentage of cell viability in the DMSO group is used as a calculation reference.

### Single-cell RNA sequencing data processing

Raw Microwell-seq data were processed following the protocols in our previously published work [2, 3]. The digital gene expression (DGE) data matrices were obtained using the Drop­seq core computational protocol (<http://mccarrolllab.org/dropseq/>). Read pairs with barcode base quality of less than 10 were removed and the remaining reads were aligned to the *Homo sapiens* GRCh38 genome using STAR [4]. The DGE data were filtered out cells with the detection of < 500 transcripts and 200 genes, as well as cells with a high proportion of mitochondria-encoded genes. The potential doublets were removed from each individual library using the R package DoubletFinder [5]. After obtaining the processed DGE data, Scanpy [6] and Seurat [7] were used for dimension reduction, clustering, and differential gene expression analysis.

The DGE of each sample was merged and fed to Scanpy for clustering. Briefly, after normalization and log transformation, 3,000 highly variable genes were selected for downstream analysis, and 60 PCs were chosen for PCA and computed the neighborhood graph of cells. Leiden algorithm was used for clustering with a resolution of 2.0, t-distributed Stochastic Neighbor Embedding (*t*-SNE) was applied for visualization, and the Wilcoxon rank-sum test was used for calculating differentially expressed genes in each cluster. We annotated each cell type by marker genes with extensive literature reading. For individual patient or single-lineage analyses, the DGE was analyzed in Seurat, including selecting variable genes, reducing dimensionality, clustering the cells, searching for differentially expressed genes, and visualizing by Uniform Manifold Approximation and Projection (UMAP) method.

### Malignant cell identification

Malignant cells were identified simultaneously using three methods. First, the DGE data of each patient were merged and clustered using the Seurat pipeline. Based on the differentially expressed genes, clusters were annotated in major five lineages, including epithelial (expressing *EPCAM*, *KRT*), endothelial (*ENG*, *VWF*, *PLVAP*), stromal (*DCN*, *COL1A1*), lymphoid (*CD3D*, *CD8A*, *FOXP3*, *MS4A1*, *IGKC*, *JCHAIN*), myeloid (*CD1C*, *IL1B*, *C1QA*, *S100A8*, *TPSAB1*). For epithelial cell types, the expression patterns of each cluster were examined to distinguish potential malignant, genes that were overexpressed in malignant relative to normal tissue for each cancer type were examined. For example, *VDAC1* [8] for BC, *RNF43* [9] for CRC, *GPC3* [10] for HCC, *CD151* [11] for ICC, *CEACAM6* [12] for LUAD, and *LAMC2* [13] for PDAC. Second, RNA-based copy-number variation inference was performed on all epithelial cells for each patient in the inferCNV [14] package, using epithelial cells of corresponding normal tissues of the cancer type in HCL as a reference. Compared to normal epithelial cells from HCL and epithelial cells from adjacent tissues, the potential malignant cells should have significantly higher inferCNV scores. Third, dimensionality reduction was performed on the potential malignant cells from different cancer types, which should form separate clusters [15].

### Cellular communication analysis

CellphonedDB [16] was used to perform cell–cell interactions among all cell lineages and in individual patients. Normalized and log-transformed data and annotation of cell types were used as input files, running with the statistical method and default parameters. The original heatmap plot from CellPhoneDB and the Circlize [17] package were used to display the frequency of interactions between two cell subsets, the ‘plot_cpdb’ in the ktplotspy package for visualizing ligand–receptor interactions.

### Gene sets enrichment analysis

Hallmark pathway analyses were performed by gene set variation analysis (GSVA) [18]. The normalized average expression of each cluster and ‘hallmark’ gene sets collected from the Molecular Signatures Database (MSigDB) [19] were used as input files. The ‘ssgsea’ method of GSVA was used to assess the EMT scores of different clusters, and the signature genes of EMT obtained from MSigDB. The clusterProfiler [20] package was used for functional enrichment analysis with default settings. Metabolism signature enrichment analysis of scRNA-seq was performed using scMetabolism [21] with default parameters. Analysis of Variance (ANOVA) was used to perform differential analysis.

### Single-cell regulatory network inference and clustering (SCENIC) and regulatory network analysis

pySCENIC [22, 23] was used to infer the regulatory network of transcription factors, with pseudo-bulk expression of the normalized data as input. The regulon enrichment of different clusters was identified using “aucell” method and the results from the RSS score were visualized using the pheatmap package. To construct gene regulatory networks that display the relationships between TFs and their target genes, the top 10 significantly specific TFs for each cluster and the top 10 target genes corresponding to these TFs were obtained from SCENIC results. To visualize the network, a custom script was employed to designate TFs or genes as nodes and the connections between TFs and genes as edges. Finally, the network was visualized using Cytoscape, and the display style was derived from SCENIC+ [24].

### Data processing for deep learning

For perturbation data, the level 5 data of CMap 2020 was downloaded from CLUE (<https://clue.io/releases/data-dashboard>), which consisted of ~8 billion gene-expression profiles on the responses of ~240 human cell lines to over ~39,320 compounds across a range of concentrations. Then the strict criteria were used to select high-confidence signatures (sigmetrics), including high technical and functional quality, high transcriptional activity score, and high correlations with replicates (is_hiq = 1, tas > 0.2, cc_q75 >= 0.2, pct_self_rank_q25 < 0.05, is_exemplar_sig = 1, nsample >=3). 8,642 high-confidence signatures were obtained. The corresponding gene-expression profiles for the high-confidence signatures were selected with level 5 data and then sorted for each signature. The top 10% significantly differentially highly expressed or low expressed genes for each signature were selected respectively and saved in gmt format. Each line in this file corresponds to a term, which represents a unique and specific gene set associated with particular compounds and experimental conditions in our study. 17,284 terms (signature conditions) with significantly differentially expressed features (genes) were obtained, i.e., the perturbed data after preprocessing.

The dataset was divided into the training set and the prediction set for deep learning. The cell types in the single-lineage analyses were randomly split, 42,517 cells from 8 cell types (about 10%) were left out for prediction, and the remaining cells were used for training. For 10-fold cross-validation analysis, cells were randomly split into 10 identical parts according to cell types in the single-lineage analyses. Randomly selecting 1 part for prediction and the remaining for training, repeating 10 times using different training and prediction sets.

By processing CMap data and the pan-cancer landscape, we constructed high-quality preprocessed perturbation data and training datasets for deep learning. The data labels can be accessed at GitHub.

### Shennong analytical workflow

The Shennong framework consists of three main stages (Fig. 3A). In the first stage, the merged scRNA-seq count matrix was integrated with the preprocessed perturbation data to obtain perturbation change features. The binary matrix was used to present whether the significantly differentially expressed features of each term were expressed or not in the scRNA-seq data. In the binary matrix, the rows and columns represent terms and cells, respectively.

In the second stage, Shennong established a cellular perturbation predictive model to capture cellular responses to pharmacological perturbations at the single-cell level. The variational autoencoder architecture was adopted to incorporate individual cells from different conditions and ensure full capture of term variability, based on publicly available model expiMap [25]. The model contained 4 hidden layers for the encoder network and the same layers in reversed order for the decoder network, with non-linear encoder for flexibility and masked linear decoder for interoperability. In the bottleneck network, the latent space dimension was equal to term numbers. The model was trained on reference scRNA-seq data and perturbation data. The scRNA-seq count matrix was received with the different conditions labels encoded by an additional vector for each cell and the binary matrix of merged perturbation data was encoded by a set of latent variables representing terms. In this study, conditions indicated that cells originated from tumor, adjacent, or normal tissues. Considering large scale of perturbation data and potential redundant perturbation information for some cells, the attention-like mechanism was implemented within the group lasso regularization in latent space to select perturbation influence terms on each cell. After pruning and enriching, the terms were fed into a linear decoder. The term matrix was used to program the neural network architecture by wiring the model parameters of the decoder to learn a specific term for each latent dimension.

In the third stage, the influence induced by each term on each cell was measured based on the trained end-to-end model. In prediction, the latent representation of the training and the prediction sets to the same latent space while correcting for batch effects between them. The influence scores of terms in each cell were measured by capturing both the latent scores and directions, stored in a matrix. Differential terms for cell clusters were identified at the term level using a Bayes test. A term was considered significantly differential in a cell type if the absolute logarithmic Bayes score for that term was greater than or equal to 2.3, referred to as the enrichment score. The resulting enrichment score matrix had each row representing a cell cluster, while each column indicated the influence of a term across the clusters. The absolute values of decoder weights for genes in each term were extracted and ranked to measure the genetic contributions of each term. The higher the absolute value of the weight meant that this gene was affected more by the term, reflecting the comparative importance of the gene in the term.

### Training

During the training phase, we simultaneously employed reconstruction loss and kullback-leibler (kl) divergence as objective functions to quantify the discrepancies between each prediction and the corresponding target at each epoch. The learning rate was employed 1e-3. An early stopping mechanism was implemented to conclude training when the minimum training loss did not decrease for 50 consecutive epochs to reduce the risk of overfitting. All models were implemented using scArches [26], PyTorch (<https://pytorch.org>), CUDA 12.3, and the Anaconda3 distribution of Python and trained on a computer cluster equipped with four NVIDIA RTX A4000 GPUs. The training phase was achieved in ~8 hours, with computational times varying depending on the dataset size and GPU device used.

### Application of Shennong to the pan-cancer landscape

In this work, we applied the Shennong framework to the pan-cancer landscape. The pan-cancer landscape and scRNA-seq data of corresponding normal tissues from HCL were integrated, and then cells from five major lineages (epithelial, stromal, endothelial, myeloid, and lymphoid) of each patient were merged and reclustered (Fig. 2E, Extended Data Fig. 7A, and Extended Data Fig. 8A). A total of 86 cell clusters from 388,646 cells across 6 tissues, originated from tumor, adjacent, and healthy normal tissues, were collected. Of which, 346,129 cells were selected for the training set and 42,517 cells were left out for the prediction set. Both the training and prediction sets contained cells from tumor, adjacent, and normal tissues, covering five major cell lineages. The scRNA-seq count matrix of the training set was normalized, log-transformed, and searched for highly variable genes in Scanpy, and then integrated with preprocessed perturbation data to generate a perturbation binary matrix with filtering out terms with less than 12 genes. The processed training set and the perturbation matrix were fed into deep learning.

A series of pre-training to optimize the hyperparameters of the model on the training set, including the multiplies the kl divergence part of the loss, group lasso regularization coefficient, and the number and size of hidden layers. Considering the integration quality of the training, the visualization latent space was well integrated, not like individual blobs but with great integration quality, alpha_kl = 0.005 was used. The strength of group lasso regularization depends on the number of terms and dataset sizes. Considering the larger numbers of terms in preprocessed perturbation data and the numbers of scRNA-seq, alpha = 0.95 and hidden_layer_sizes=512 were used. After training on the training set, the trained weights were transferred to the prediction model with additional conditional nodes used to map the prediction set to the training set.

In prediction, features from the prediction set were extracted and mapped well to the training set (Fig. 3B and Extended Data Fig. 9B). UMAP visualization of the latent space showed that cells were divided into clusters similar to those in the single-lineage analyses. Then entire cells of pan-cancer landscape and HCL were fed into the prediction model, and the latent space visualization showed great integration. Cells from different tissue types and tissue sources were well integrated, and the cell lineages and clusters in the single-lineage analyses had distinct visualization (Extended Data Fig. 9C-D). The latent scores and directions of each cell were extracted from the prediction model with entire cells of pan-cancer landscape and HCL. The enrichment test was performed using Bayes Factors to identify significantly differential terms in different cell types. The results were saved in an influence score matrix, which reflected the effects of the compound treatment on each cell cluster. The latent scores of a term in all cells were visualized in UMAP, as well as the latent variables of two terms in all cells. The gene contributions of each term were extracted from the decoder and sorted by their absolute weight, visualized in the dot plot.

### Latent score directions

The latent scores of terms do not correspond to upregulation or downregulation of these terms, which could be determined by counting the decoder gene weights of the corresponding terms. If the majority of gene weights in the term were positive, then the higher positive latent score indicated upregulation. In the opposite case of most negative weights, the lower negative score also indicated upregulation. First, the sum of the weights of all genes in each term was calculated, indicating the potential direction of the term. Second, the raw latent scores of each term were multiplied by the direction value. Therefore, the higher positive value of this value always corresponded to the predominant upregulation of the term, while the lower negative value corresponded to downregulation.

## Reference

1. Han X, Wang R, Zhou Y *et al.* Mapping the Mouse Cell Atlas by Microwell-Seq. *Cell*. 2018; **173**(5): 1307. doi: 10.1016/j.cell.2018.05.012

2. Han X, Zhou Z, Fei L *et al.* Construction of a human cell landscape at single-cell level. *Nature*. 2020; **581**(7808): 303-309. doi: 10.1038/s41586-020-2157-4

3. Wang R, Zhang P, Wang J *et al.* Construction of a cross-species cell landscape at single-cell level. *Nucleic Acids Res*. 2023; **51**(2): 501-516. doi: 10.1093/nar/gkac633

4. Dobin A, Davis CA, Schlesinger F *et al.* STAR: ultrafast universal RNA-seq aligner. *Bioinformatics*. 2013; **29**(1): 15-21. doi: 10.1093/bioinformatics/bts635

5. McGinnis CS, Murrow LM, Gartner ZJ. DoubletFinder: Doublet Detection in Single-Cell RNA Sequencing Data Using Artificial Nearest Neighbors. *Cell Syst*. 2019; **8**(4): 329-337 e324. doi: 10.1016/j.cels.2019.03.003

6. Wolf FA, Angerer P, Theis FJ. SCANPY: large-scale single-cell gene expression data analysis. *Genome Biol*. 2018; **19**(1): 15. doi: 10.1186/s13059-017-1382-0

7. Hao Y, Hao S, Andersen-Nissen E *et al.* Integrated analysis of multimodal single-cell data. *Cell*. 2021; **184**(13): 3573-3587 e3529. doi: 10.1016/j.cell.2021.04.048

8. Shoshan-Barmatz V, Mizrachi D. VDAC1: from structure to cancer therapy. *Front Oncol*. 2012; **2**: 164. doi: 10.3389/fonc.2012.00164

9. Xu Y, Lin Z, Ji Y *et al.* Pan-cancer analysis identifies RNF43 as a prognostic, therapeutic and immunological biomarker. *Eur J Med Res*. 2023; **28**(1): 438. doi: 10.1186/s40001-023-01383-1

10. Nishida T, Kataoka H. Glypican 3-Targeted Therapy in Hepatocellular Carcinoma. *Cancers (Basel)*. 2019; **11**(9). doi: 10.3390/cancers11091339

11. Huang XY, Ke AW, Shi GM *et al.* Overexpression of CD151 as an adverse marker for intrahepatic cholangiocarcinoma patients. *Cancer*. 2010; **116**(23): 5440-5451. doi: 10.1002/cncr.25485

12. Kim EY, Cha YJ, Jeong S *et al.* Overexpression of CEACAM6 activates Src-FAK signaling and inhibits anoikis, through homophilic interactions in lung adenocarcinomas. *Transl Oncol*. 2022; **20**: 101402. doi: 10.1016/j.tranon.2022.101402

13. Kosanam H, Prassas I, Chrystoja CC *et al.* Laminin, gamma 2 (LAMC2): a promising new putative pancreatic cancer biomarker identified by proteomic analysis of pancreatic adenocarcinoma tissues. *Mol Cell Proteomics*. 2013; **12**(10): 2820-2832. doi: 10.1074/mcp.M112.023507

14. Tickle T, Tirosh I, Georgescu C *et al.* inferCNV of the Trinity CTAT Project. 2019.

15. Tirosh I, Izar B, Prakadan SM *et al.* Dissecting the multicellular ecosystem of metastatic melanoma by single-cell RNA-seq. *Science*. 2016; **352**(6282): 189-196. doi: 10.1126/science.aad0501

16. Garcia-Alonso L, Lorenzi V, Mazzeo CI *et al.* Single-cell roadmap of human gonadal development. *Nature*. 2022; **607**(7919): 540-547. doi: 10.1038/s41586-022-04918-4

17. Gu Z, Gu L, Eils R *et al.* circlize Implements and enhances circular visualization in R. *Bioinformatics*. 2014; **30**(19): 2811-2812. doi: 10.1093/bioinformatics/btu393

18. Hanzelmann S, Castelo R, Guinney J. GSVA: gene set variation analysis for microarray and RNA-seq data. *BMC Bioinformatics*. 2013; **14**: 7. doi: 10.1186/1471-2105-14-7

19. Liberzon A, Birger C, Thorvaldsdottir H *et al.* The Molecular Signatures Database (MSigDB) hallmark gene set collection. *Cell Syst*. 2015; **1**(6): 417-425. doi: 10.1016/j.cels.2015.12.004

20. Yu G, Wang LG, Han Y *et al.* clusterProfiler: an R package for comparing biological themes among gene clusters. *OMICS*. 2012; **16**(5): 284-287. doi: 10.1089/omi.2011.0118

21. Wu Y, Yang S, Ma J *et al.* Spatiotemporal Immune Landscape of Colorectal Cancer Liver Metastasis at Single-Cell Level. *Cancer Discov*. 2022; **12**(1): 134-153. doi: 10.1158/2159-8290.CD-21-0316

22. Aibar S, Gonzalez-Blas CB, Moerman T *et al.* SCENIC: single-cell regulatory network inference and clustering. *Nat Methods*. 2017; **14**(11): 1083-1086. doi: 10.1038/nmeth.4463

23. Van de Sande B, Flerin C, Davie K *et al.* A scalable SCENIC workflow for single-cell gene regulatory network analysis. *Nat Protoc*. 2020; **15**(7): 2247-2276. doi: 10.1038/s41596-020-0336-2

24. Bravo Gonzalez-Blas C, De Winter S, Hulselmans G *et al.* SCENIC+: single-cell multiomic inference of enhancers and gene regulatory networks. *Nat Methods*. 2023; **20**(9): 1355-1367. doi: 10.1038/s41592-023-01938-4

25. Lotfollahi M, Rybakov S, Hrovatin K *et al.* Biologically informed deep learning to query gene programs in single-cell atlases. *Nat Cell Biol*. 2023; **25**(2): 337-350. doi: 10.1038/s41556-022-01072-x

26. Lotfollahi M, Naghipourfar M, Luecken MD *et al.* Mapping single-cell data to reference atlases by transfer learning. *Nat Biotechnol*. 2022; **40**(1): 121-130. doi: 10.1038/s41587-021-01001-7
