# Supplementary material for: A deep learning framework for in silico screening of anticancer drugs at the single-cell level
Source: Natl Sci Rev. 2024 Dec 10;12(2):nwae451. doi: 10.1093/nsr/nwae451 (PMC11771446; doi:10.1093/nsr/nwae451)
Supplement: nwae451_Supplemental_File [file nwae451_supplemental_file.zip › Supplementary files/Supplementary files/Supplementary figure and legends.docx]

## Supplementary figure legends

**
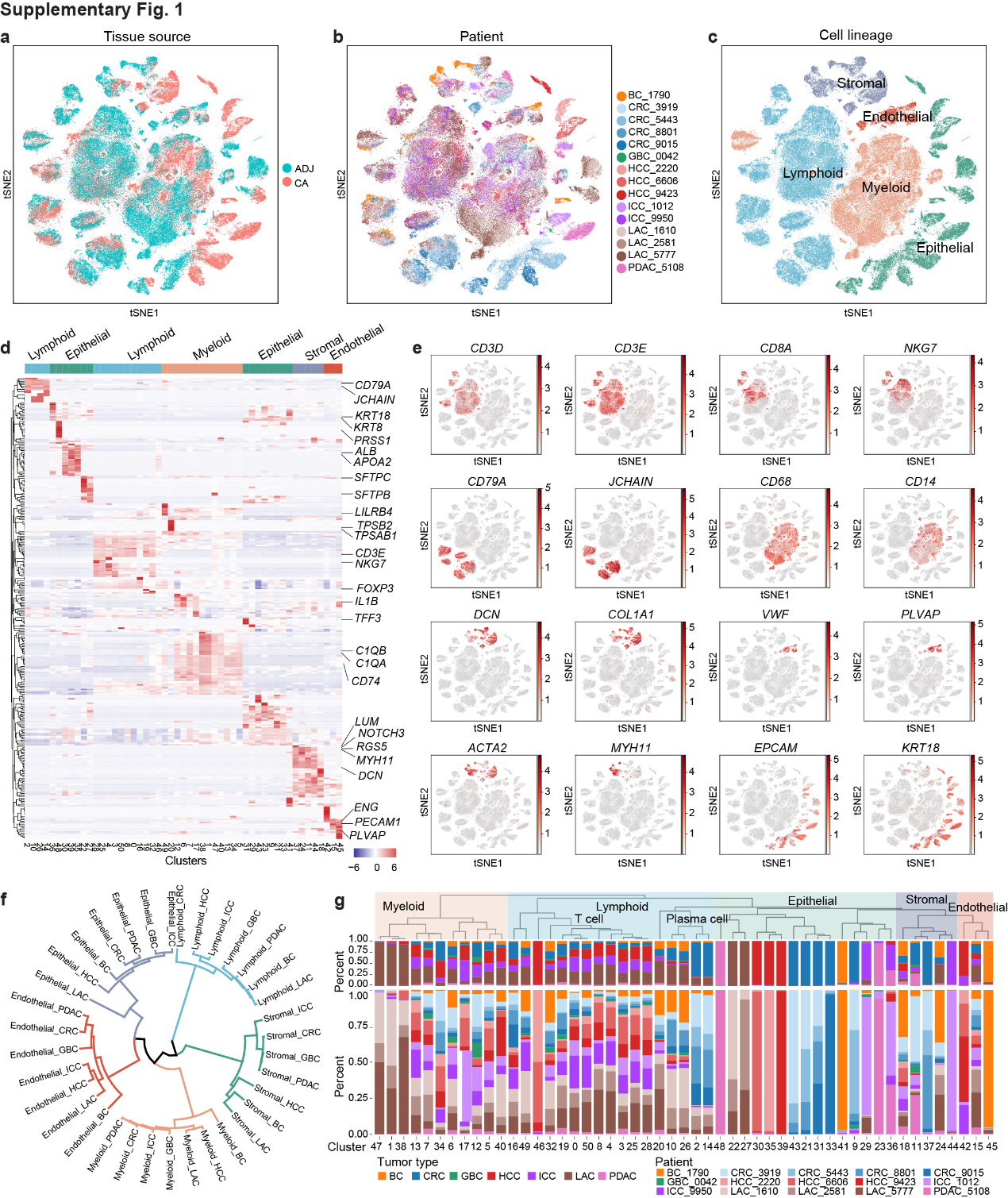
**

#### Supplementary Fig. 1. The pan-cancer cell landscape was constructed using Microwell-seq, related to Figure 1.

**(a-c)** *t*-SNE visualization of the pan-cancer landscape, colored by tissue source (A), patient (B), and cell lineage (C). **(d)** Heatmap showing the scaled expression levels of cell type-specific marker genes. **(e)** Expression patterns of representative cell lineage-specific marker genes are shown by t-SNE. **(f)** Hierarchical clustering tree showing the similarity among 5 cell lineages from different tumor types. **(g)** Hierarchical clustering tree (top) showing the similarity among 51 cell clusters, and histograms showing the percentage of tumor type (middle) and patient (bottom) for each cell cluster.


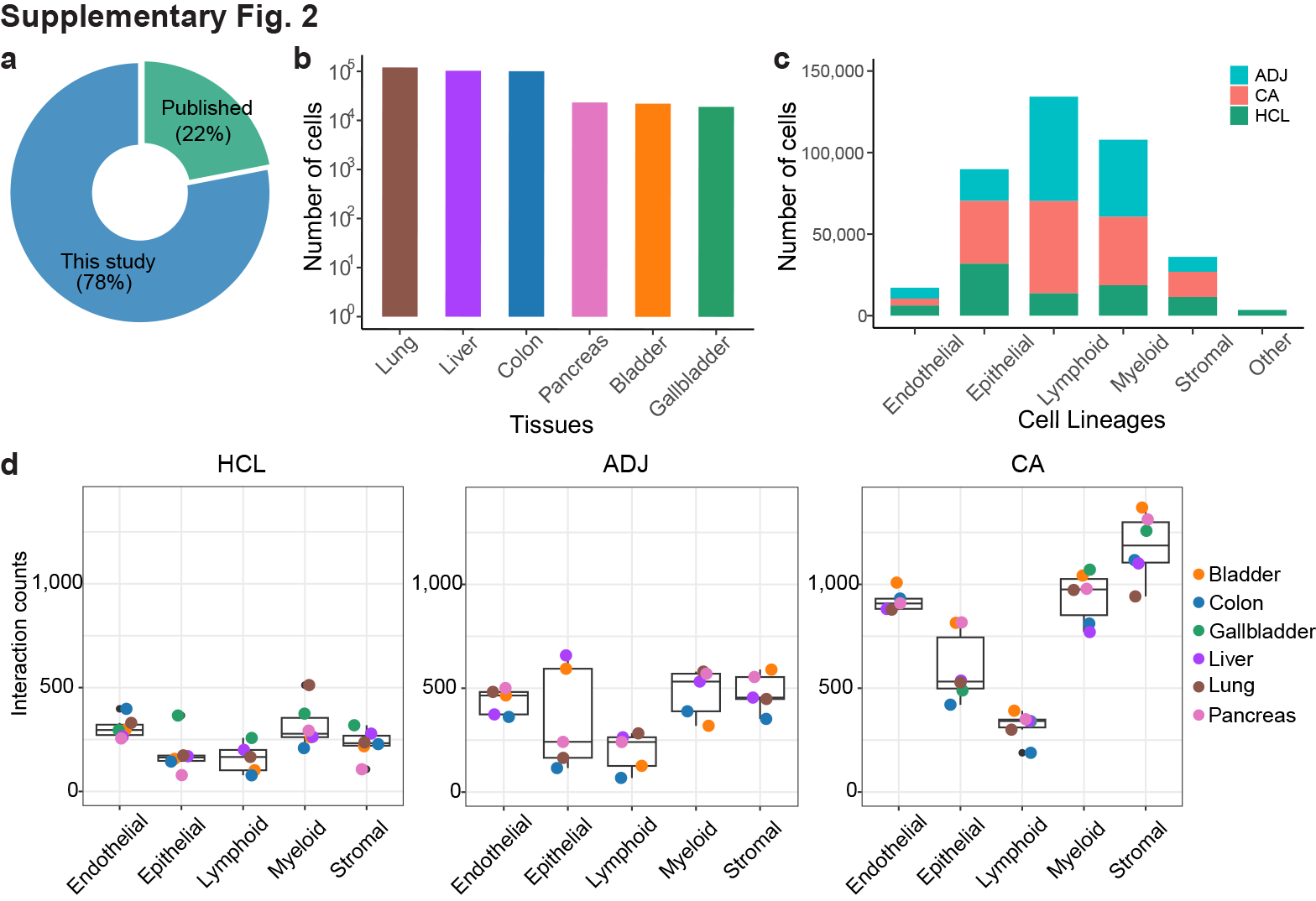


#### Supplementary Fig. 2. Cell information in the pan-cancer landscape and HCL, related to Figure 2.

**(a)** Donut chart showing percentage of analyzed cells in this study (pan-cancer landscape, CA/ADJ tissues) and our previous work (HCL, normal tissues). **(b)** Number of analyzed cells per tissue. **(c)** Stacked bar chart showing the number of analyzed cells from each tissue source and each cell lineage. **(d)** Count of cellular interactions among 5 main cell lineages in TME components from different tissue source.


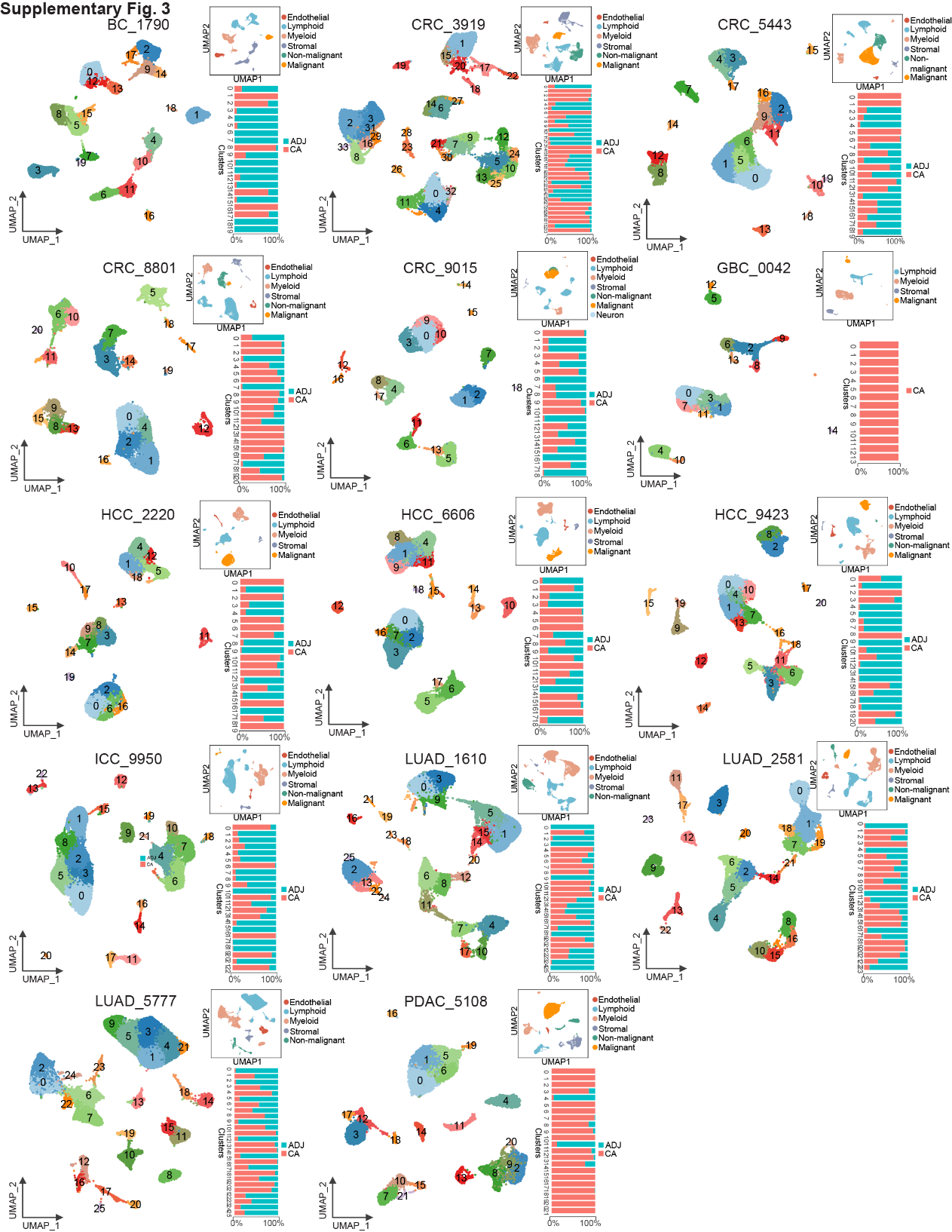


#### Supplementary Fig. 3. Visualization and composition of cell clusters for each patient in the pan-cancer landscape, related to Figure 2.

UMAP visualization of cells clusters (left) and cell lineages (top right) for the patient. Bar plot (bottom right) showing the percentage of tumor source for each cell cluster.

**
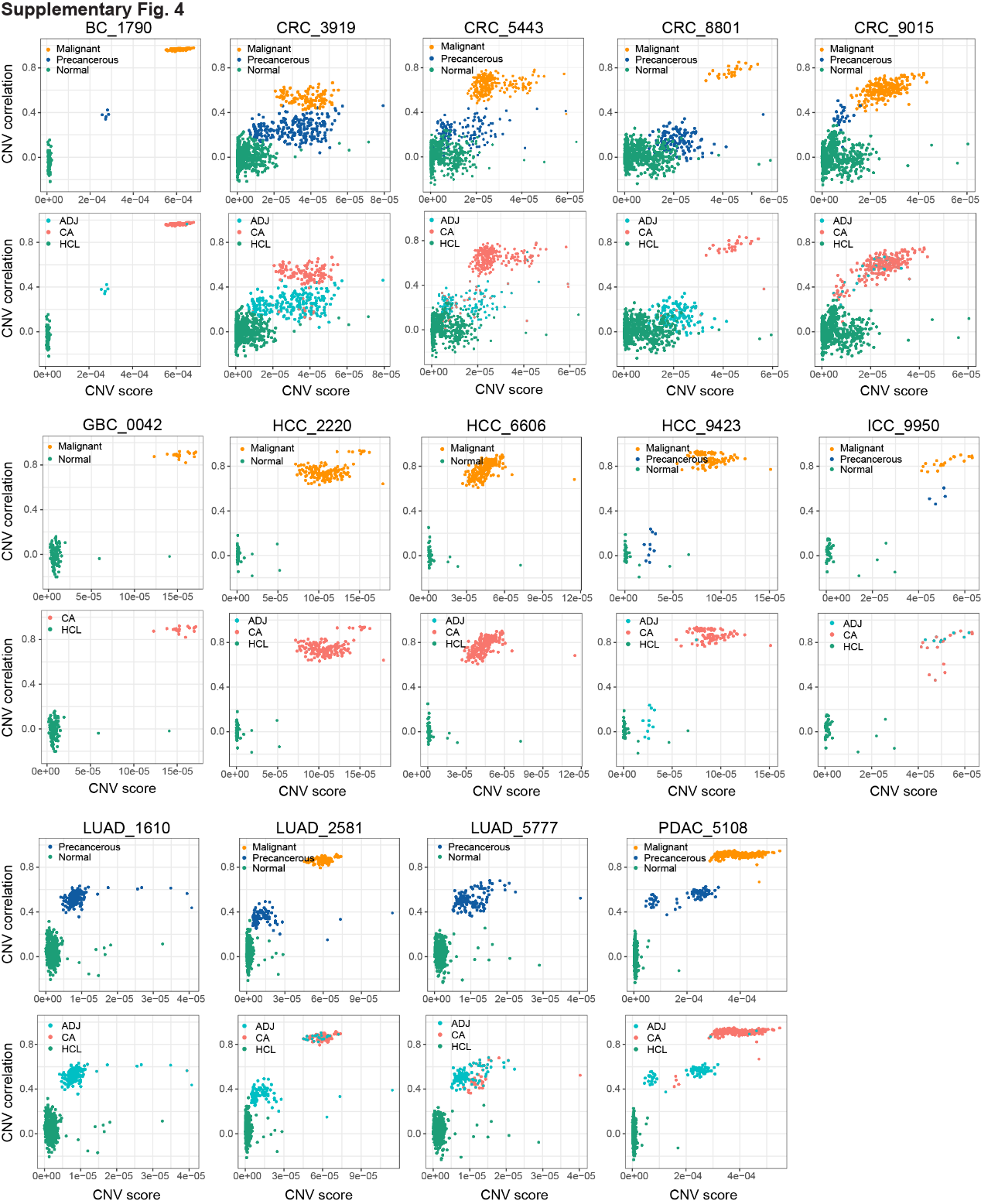
**

#### Supplementary Fig. 4. Visualization of inferring CNVs for each patient in the pan-cancer landscape, related to Figure 2.

Malignant type classification (top) and tissue source distribution (bottom) of inferred CNV scores (x-axis) and CNV correlations (y-axis) for epithelial cells in each patient.


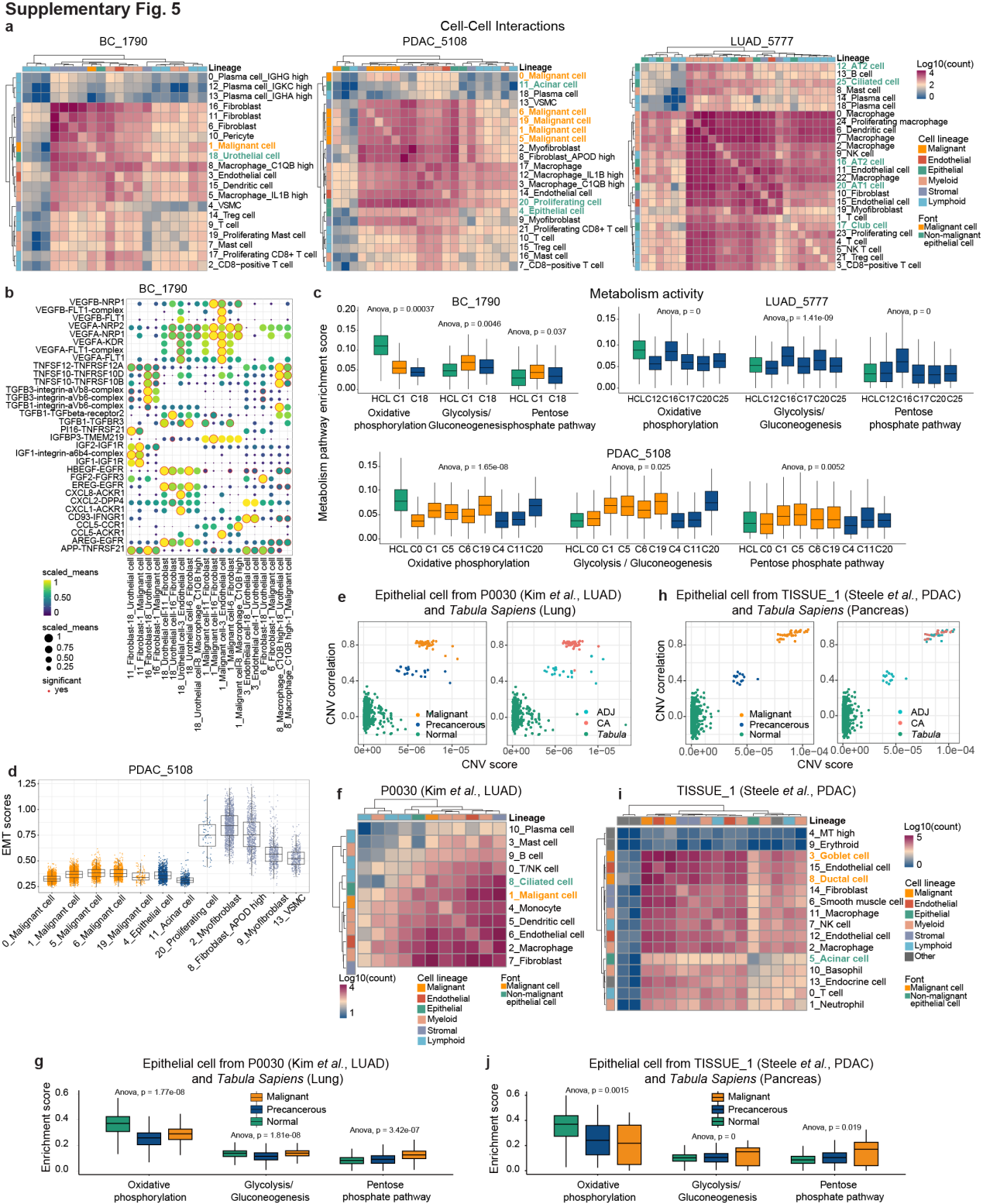


#### Supplementary Fig. 5. Identification of malignant and precancerous cells via single-sample analyses, related to Figure 2.

**(a)** The cell interactions between cell clusters for patient BC_1790, PDAC_5108, and LUAD_5777. Malignant cell types are colored orange and non-malignant epithelial cell types are colored green. **(b)** Dot plot showing the ligand-receptor enrichment of cell clusters in patient BC_1790. **(c)** Boxplot showing enrichment scores of ‘oxidative phosphorylation’, ‘glycolysis/gluconeogenesis’ and ‘pentose phosphate pathway’ metabolism pathway in epithelial clusters of corresponding normal tissue and patients BC_1790, PDAC_5108, and LUAD_5777. **(d)** Box plot showing EMT scores in epithelial and stromal cell clusters from patient PDAC_5108. **(e, h)** Malignant type classification and tissue source distribution of inferred CNV scores (x-axis) and CNV correlations (y-axis) for epithelial cells in P0030 (left, Kim *et al.*, LUAD) and TISSUE_1 (right, Steele *et al.*, PDAC). **(f, i)** The cell interactions between cell types for patients P0030 (left) and TISSUE_1 (right). **(g, j)** Boxplot showing enrichment scores of ‘oxidative phosphorylation’, ‘glycolysis/gluconeogenesis’ and ‘pentose phosphate pathway’ metabolism pathway in epithelial cell types of corresponding normal tissue and patients P0030 (left) and TISSUE_1 (right).


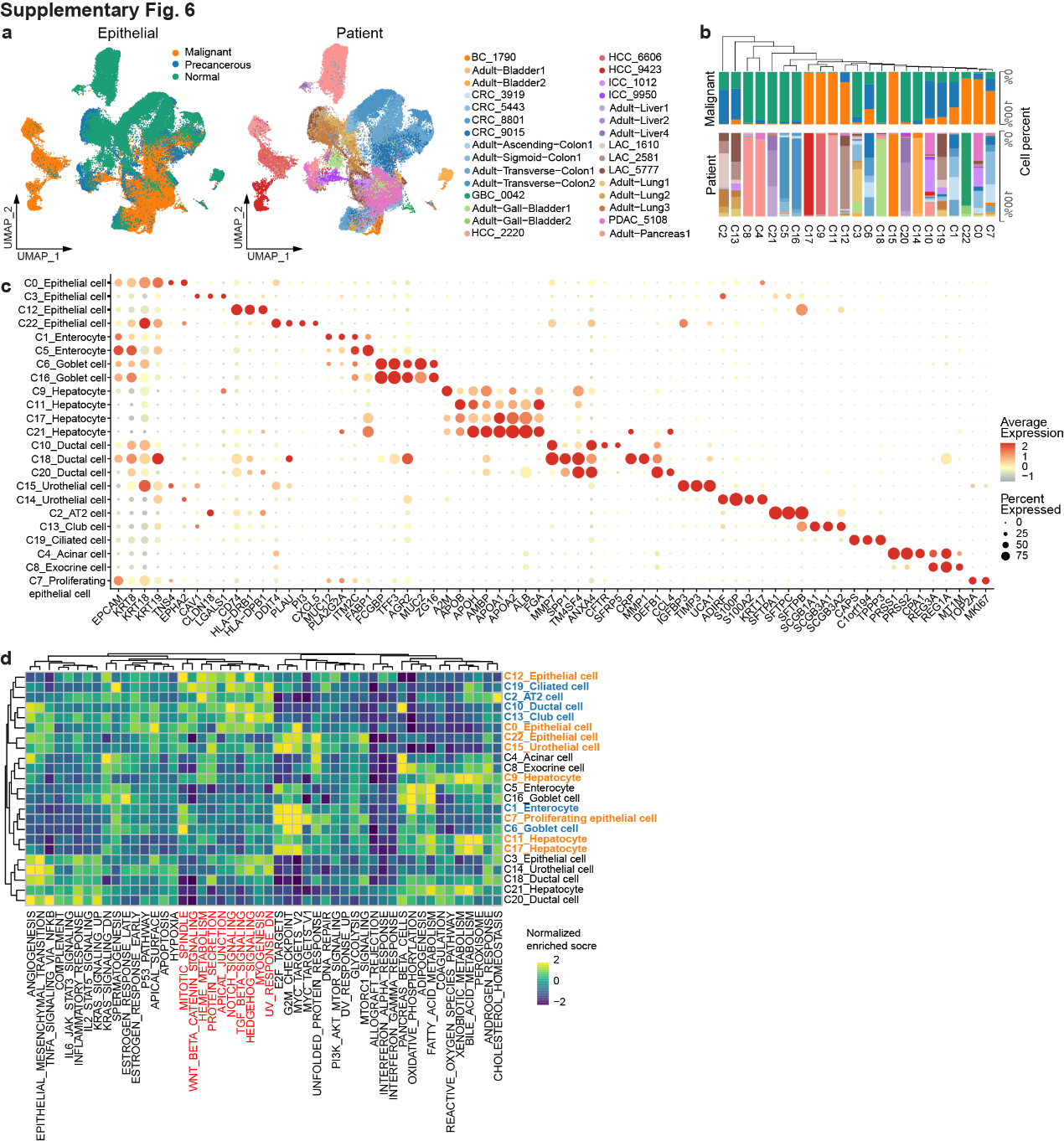


#### Supplementary Fig. 6. Profiling malignant and precancerous cells via pan-cancer analyses, related to Figure 3.

**(a)** UMAP visualization of epithelial cells from the pan-cancer and HCL, colored by malignant type classification (left) and patient (right). **(b)** Hierarchical clustering tree (top) showing the similarity among 23 epithelial cell clusters, and histograms showing the percentage of malignant type (middle) and patient (bottom) for each cell cluster. **(c)** Dot plots showing scaled average expression levels of cell type-specific markers in 23 epithelial cell clusters. **(d)** Hallmark gene ontology enrichment of 23 epithelial cell clusters.


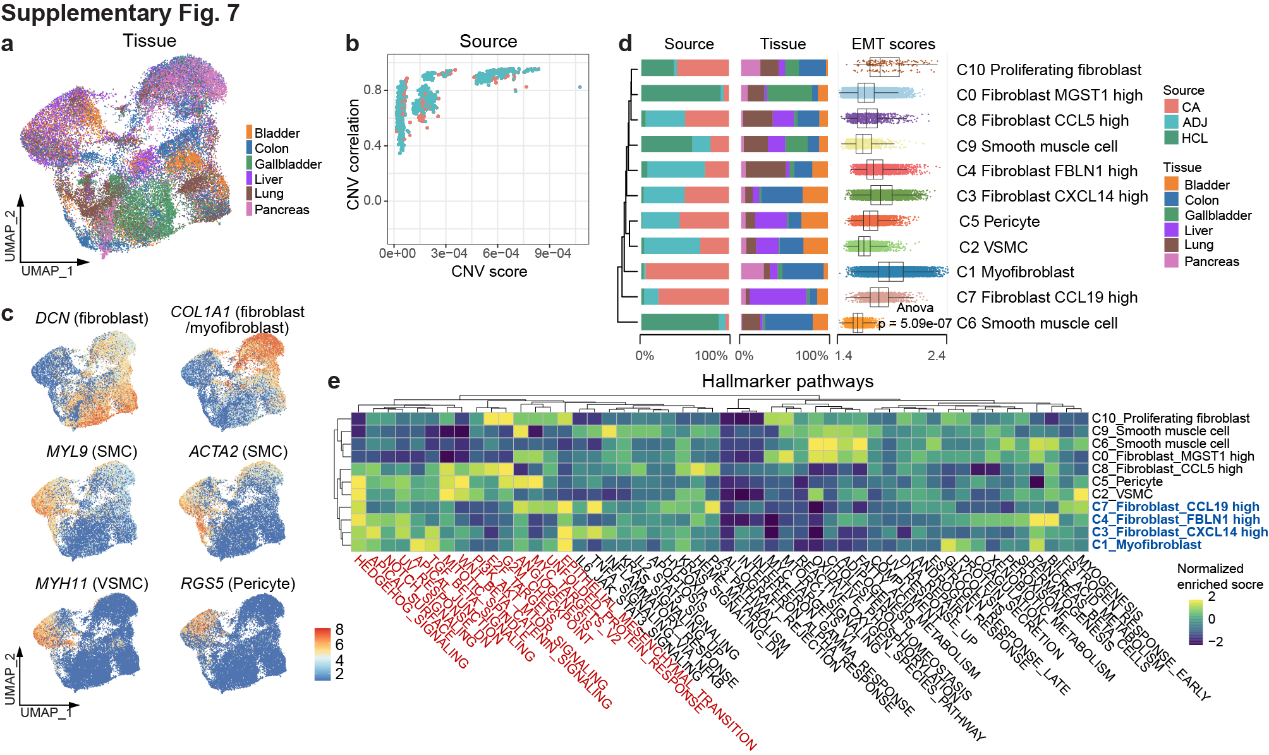


#### Supplementary Fig. 7. Profiling stromal cells via pan-cancer analyses, related to Figure 3.

**(a)** UMAP visualization of all stromal cells from the pan-cancer landscape and HCL, colored by tissue type. **(b)** Tissue source distribution of inferred CNV scores (x-axis) and CNV correlations (y-axis) for all stromal cells. **(c)** UMAP visualization of all stromal cells, colored by cell type representative markers. **(d)** Bar plot showing the percentage of tissue source (left), tissue type (middle), and EMT score (right) for each stromal cluster. **(e)** Hallmark gene ontology enrichment of 11 stromal cell clusters.


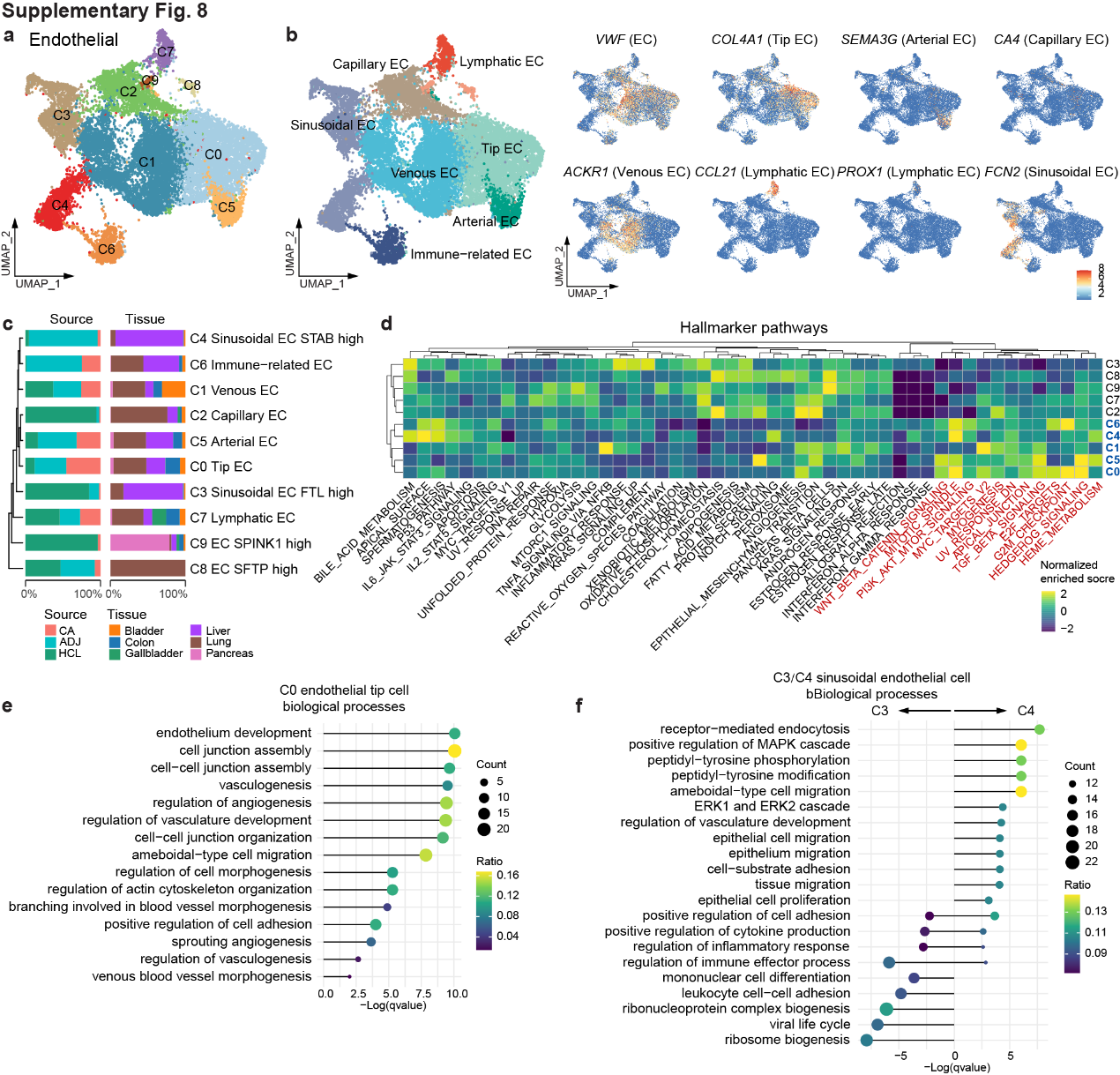


#### Supplementary Fig. 8. Profiling endothelial cells via pan-cancer analyses, related to Figure 3.

**(a)** UMAP visualization of clusters (n=10) for all endothelial cells from the pan-cancer landscape and HCL. **(b)** UMAP visualization of all endothelial cells, colored by main cell type (left) and cell type representative markers (right). **(c)** Bar plot showing the percentage of tissue source (left) and tissue type (middle) for each endothelial cell cluster. **(d)** Hallmark gene ontology enrichment of 10 endothelial cell clusters. **(e)** Gene ontology enrichment of differentially expressed genes in biological processes in C0 endothelial tip cells. **(f)** Gene ontology enrichment of differentially expressed genes in biological processes in C3/C4 sinusoidal endothelial cells.


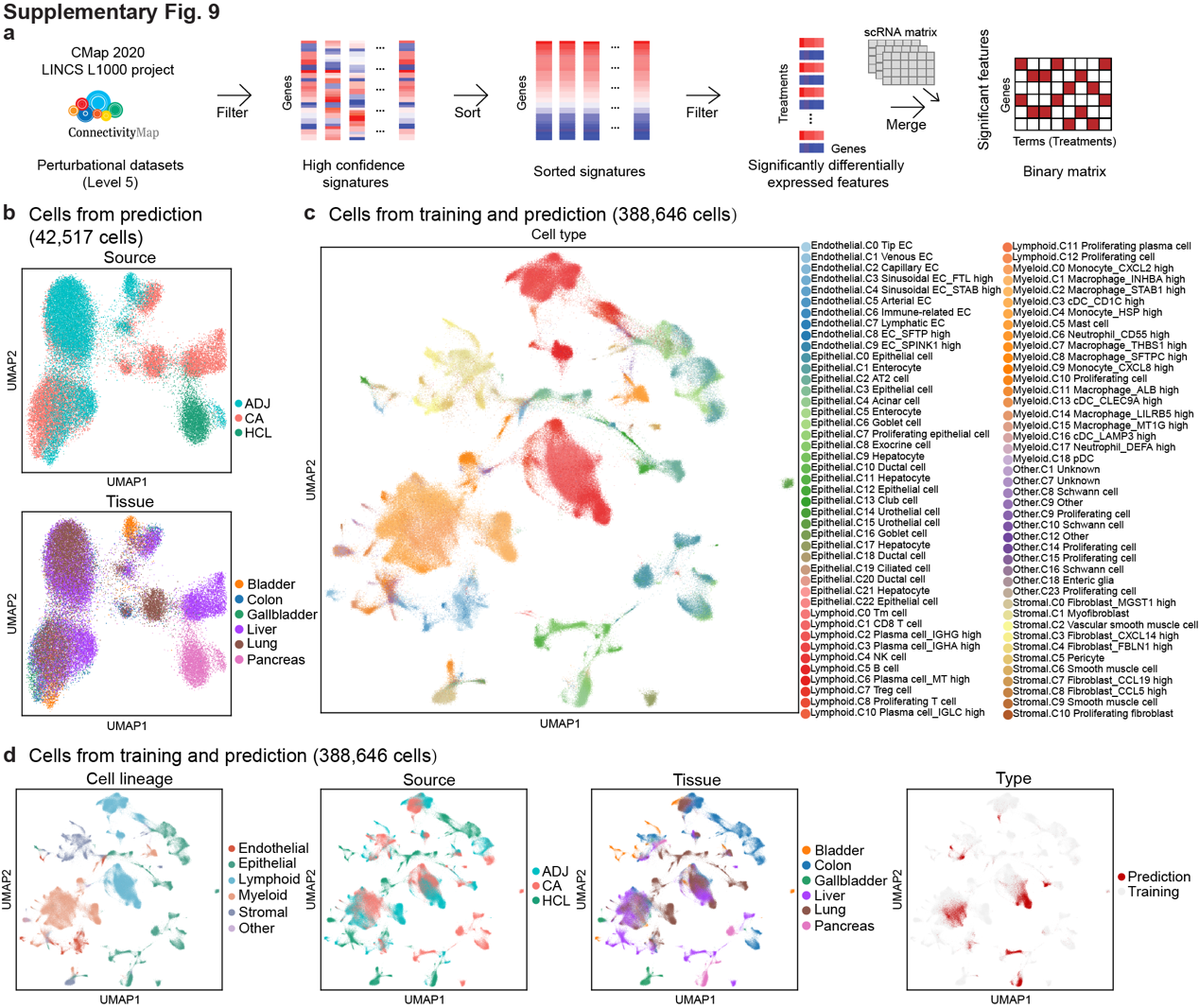


#### Supplementary Fig. 9. Interpretable single-cell level drug perturbation prediction using Shennong, related to Figure 4.

**(a)** Workflow for perturbation datasets preprocessing. **(b)** UMAP representation of the prediction set (n=42,517 cells) embedded in latent space extracted from the framework, colored by tissue source (top) and tissue type (bottom). **(c)** UMAP representation of all the cells in the pan-cancer landscape and HCL (n=388,646 cells) embedded in latent space extracted from the framework, colored by cell type. **(d)** UMAP representation of all the cells in the pan-cancer landscape and HCL (n=388,646 cells) embedded in latent space extracted from the framework, colored by cell lineage, tissue source, tissue type, and set type (from left to right).

**
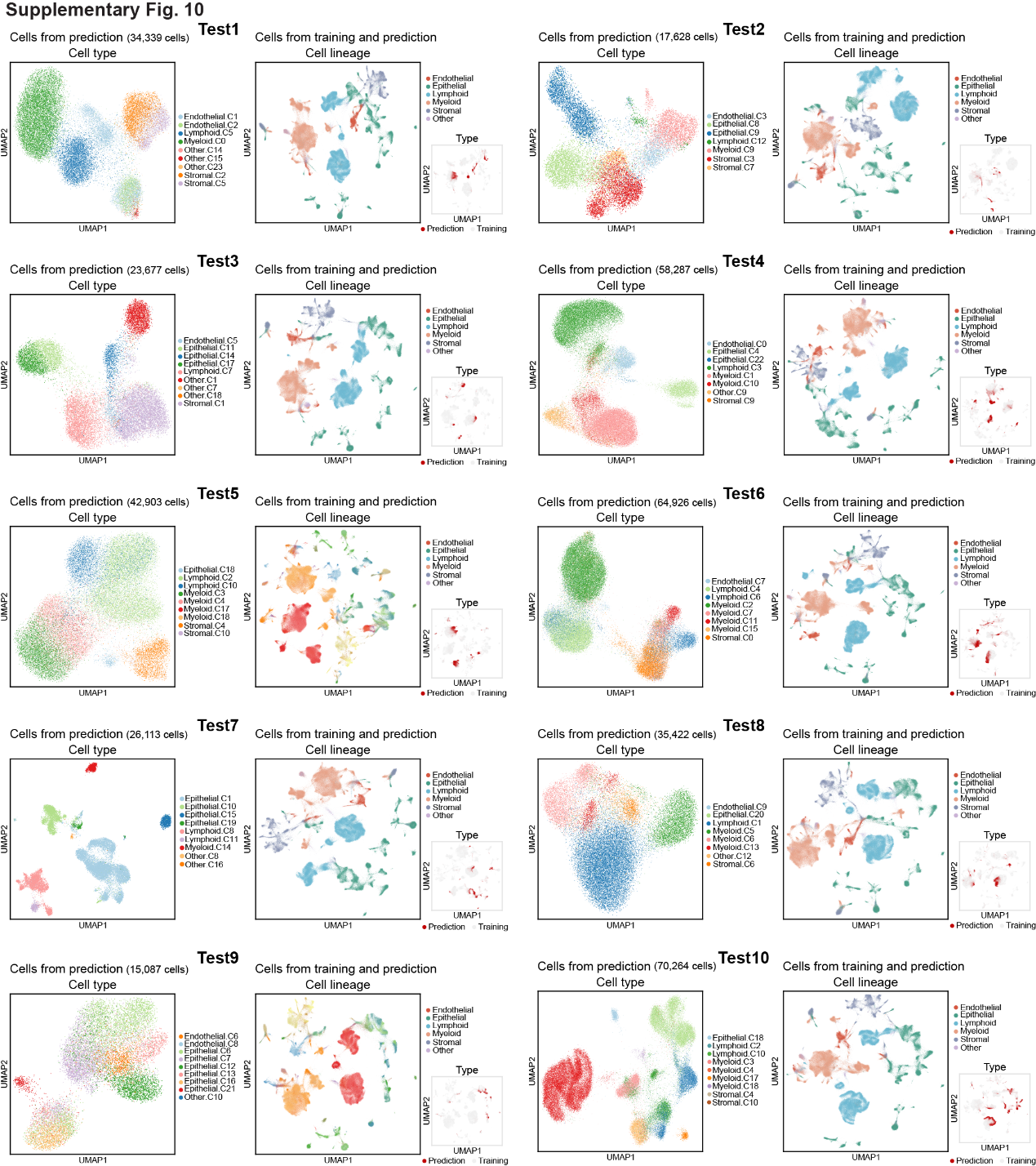
**

#### Supplementary Fig. 10. 10-fold cross-validation of the Shennong framework, related to Figure 4.

The 10-fold cross-validation analysis was performed on all cells from the pan-cancer landscape and HCL using Shennong. In each validation, UMAP representation of the prediction set and all cells embedded in latent space extracted from the framework, colored by cell type (left), cell lineage (middle), and set type (right).


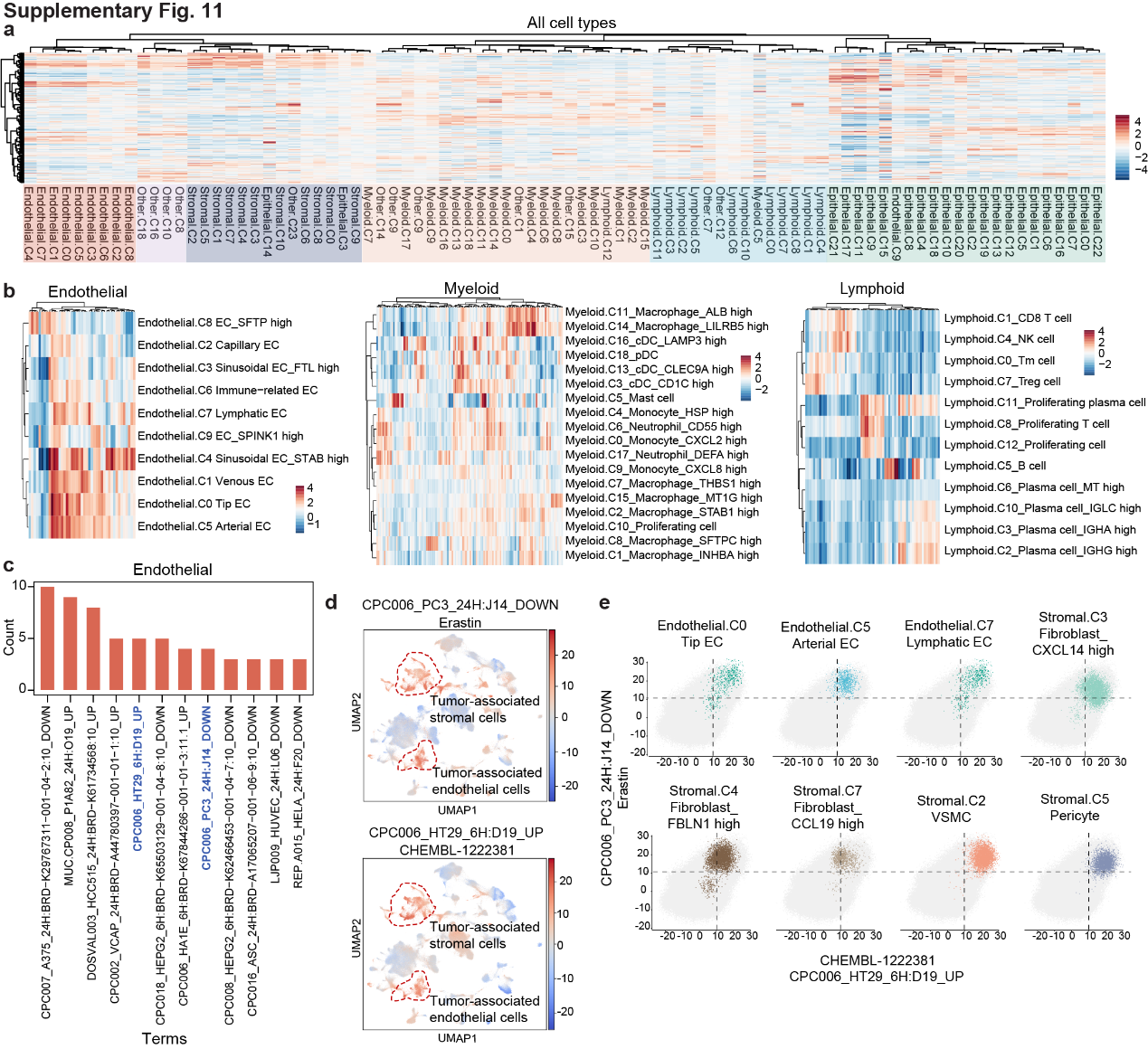


#### Supplementary Fig. 11. Interpretable single-cell level drug perturbation prediction using Shennong, related to Figure 4.

**(a)** Heatmaps showing the scaled influence term scores of top 10 significantly differential terms in all cell types in the single-lineage analyses. **(b)** Heatmaps showing the scaled influence term scores of top 10 significantly differential terms in endothelial (left), myeloid (middle), and lymphoid cells (right). **(c)** Bar plot showing counts of top 10 significantly differential terms in each cell type in endothelial lineage. Terms that are further analyzed are colored blue. **(d)** UMAP representation of the influence term scores of all cells for terms CPC006_PC3_24H:J14_DOWN (top) and CPC006_HT29_6H:D19_UP (bottom), corresponding to the compounds erastin and CHEMBL-1222381, respectively. **(e)** Visualization of each tumor-associated stromal or endothelial cell types (from left to right) in the context of the terms mentioned in D. Each dot shows the influence terms score of each cell.


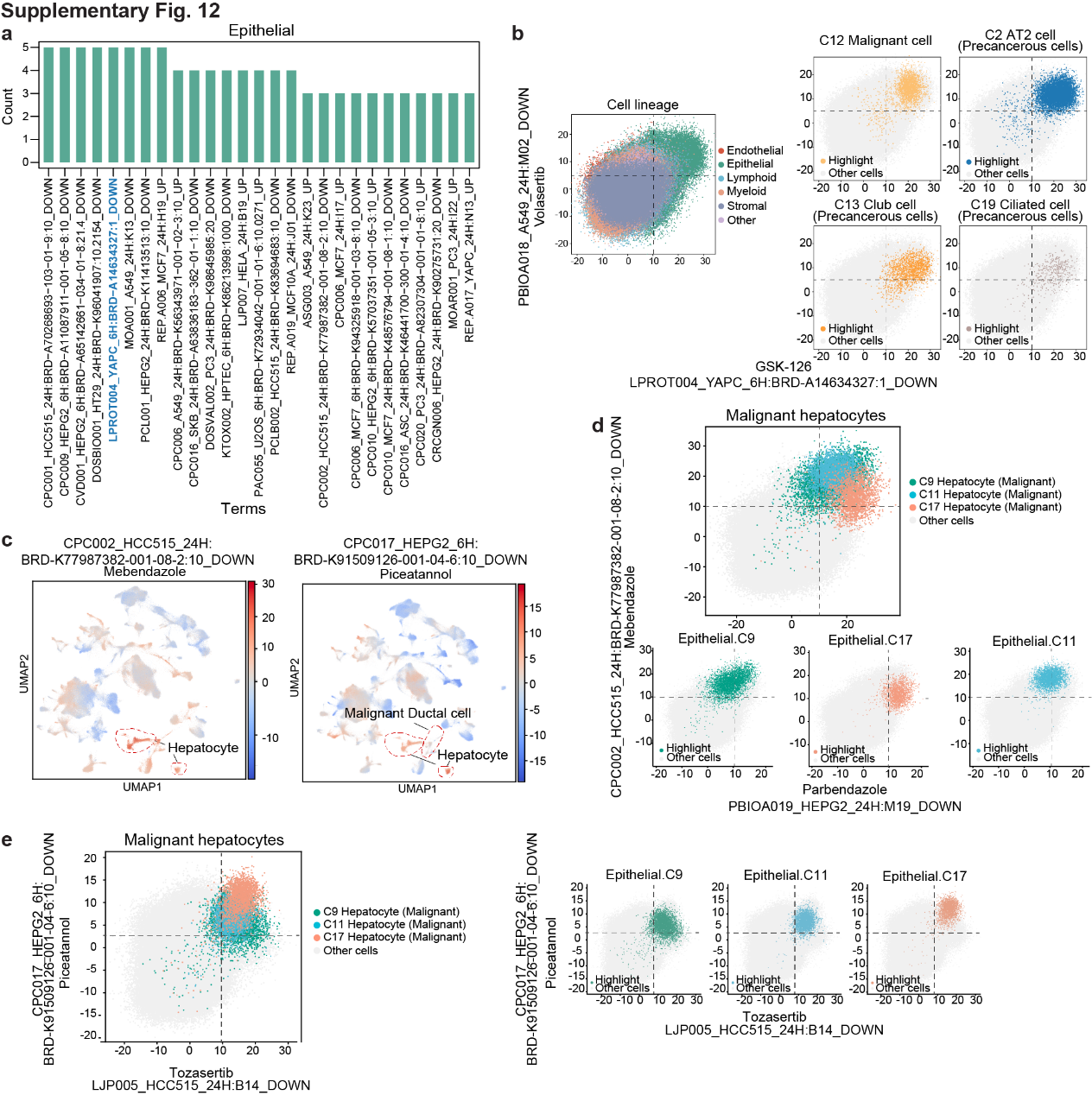


#### Supplementary Fig. 12. Interpretable single-cell level drug perturbation prediction in epithelial using Shennong, related to Figure 5.

**(a)** Bar plot showing counts of top 10 significantly differential terms in each cell type in epithelial lineage. Terms that are further analyzed are colored blue. **(b)** Visualization of cell lineages (left) and each malignant and precancerous cell types mainly from lung (right) in the context of terms LPROT004_YAPC_6H:BRD-A14634327:1_DOWN and PBIOA018_A549_24H:M02_DOWN, corresponding to the compounds GSK-126 and volasertib, respectively. Each dot shows the influence terms score of each cell. **(c)** UMAP representation of the influence term scores of all cells for terms CPC002_HCC515_24H:BRD-K77987382-001-08-2:10_DOWN (left), and CPC017_HEPG2_6H:BRD-K91509126-001-04-6:10_DOWN (right), corresponding to the compounds mebendazole and piceatannol, respectively. **(d)** Visualization of malignant hepatocytes (top) and each cell type of malignant hepatocytes (bottom) in the context of the terms PBIOA019_HEPG2_24H:M19_DOWN and CPC002_HCC515_24H:BRD-K77987382-001-08-2:10_DOWN. **(e)** Visualization of malignant hepatocytes (left) and each cell type of malignant hepatocytes (right) in the context of the terms LJP005_HCC515_24H:B14_DOWN and CPC017_HEPG2_6H:BRD-K91509126-001-04-6:10_DOWN. Each dot shows the influence terms score of each cell.


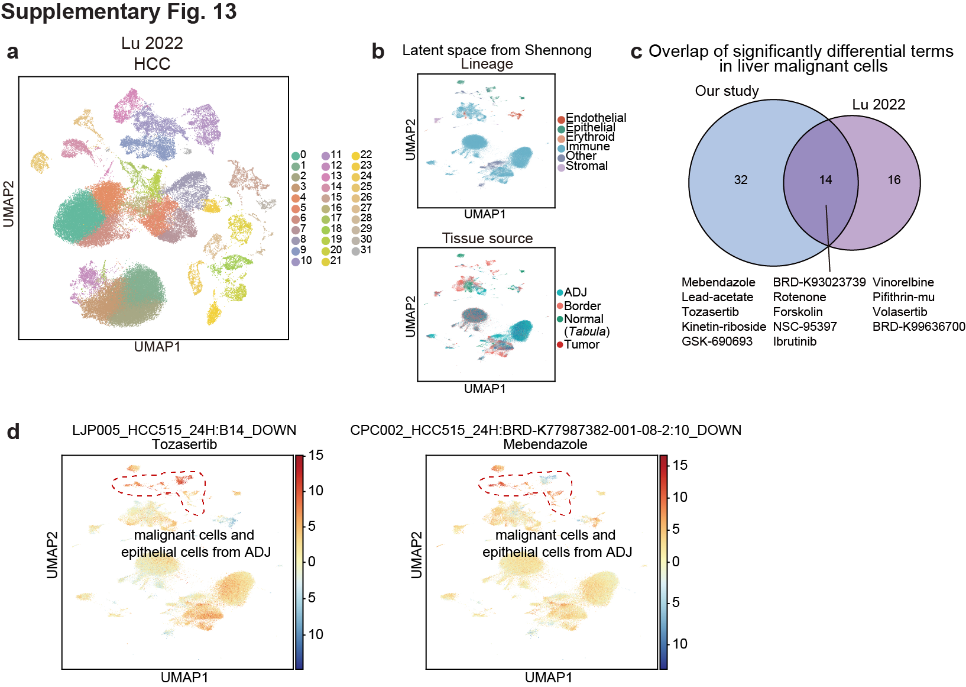


#### Supplementary Fig. 13. Comparisons of the prediction results between our pan-cancer landscape and third-party datasets, related to Figure 5.

**(a)** UMAP visualization of cells from the HCC dataset (75,991 cells), colored by cell clusters. **(b)** UMAP representation of cells in the HCC dataset and liver tissue from Tabula Sapiens (n=80,998 cells) embedded in latent space extracted from the framework, colored by cell lineage (top) and tissue source (bottom). **(c)** Overlap of significantly differential terms in liver cancer malignant cells between pan-cancer landscape (C9, C11, and C17) and the third-party HCC dataset (clusters 18 and 27). Only terms observed in at least two clusters in each dataset were counted. **(d)** UMAP representation of influence term scores of all cells for terms LJP005_HCC515_24H:B14_DOWN (left), CPC002_HCC515_24H:BRD-K77987382-001-08-2:10_DOWN (right), corresponding to the compounds tozasertib and mebendazole, respectively.


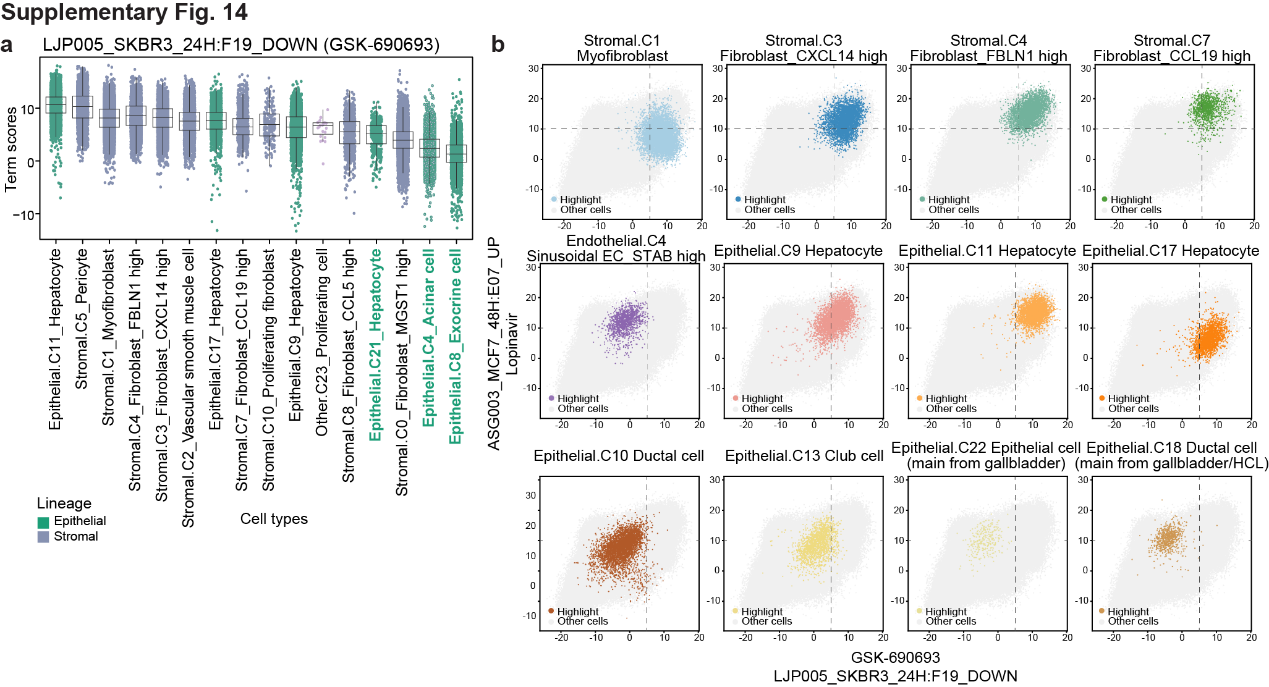


#### Supplementary Fig. 14. Tissue damaging effects prediction of anticancer drugs using Shennong, related to Figure 6.

**(a)** Box plot showing influence term scores of top 16 cell types in the term LJP005_SKBR3_24H:F19_DOWN corresponding to the compound GSK-690693. Epithelial cell types originating from HCL were colored green. **(b)** Visualization of each tumor-associated fibroblasts, malignant and precancerous epithelial cell types mentioned in C in the context of the terms LJP005_SKBR3_24H:F19_DOWN and ASG003_MCF7_48H:E07_UP, corresponding to the compounds GSK-126 and volasertib, respectively. Each dot shows the influence terms scores of each cell.
