# Supplementary material for: A deep learning framework for in silico screening of anticancer drugs at the single-cell level
Source: Natl Sci Rev. 2024 Dec 10;12(2):nwae451. doi: 10.1093/nsr/nwae451 (PMC11771446; doi:10.1093/nsr/nwae451)
Supplement: nwae451_Supplemental_File [file nwae451_supplemental_file.zip › Supplementary files/Supplementary files/Supplementary tables list.docx]

## Supplementary tables

**Supplementary Table S1.** Clinical characteristics of patients.

**Supplementary Table S2.** Metadata of profiled samples, includes individual collagenase type and digestion time for all profiled tissue samples.

**Supplementary Table S3.** Indexed bead sequences and oligonucleotide sequences used in beads synthesis.

**Supplementary Table S4.** Oligonucleotide sequences used in Microwell-seq.

**Supplementary Table S5.** Sequencing information of profiled samples.

**Supplementary Table S6**. Differentially expressed genes for 51 clusters in the pan-cancer single-cell landscape. Adjusted p values were calculated using two-sided Wilcoxon rank-sum test.

**Supplementary Table S7**. Cell type annotations for 51 clusters in the pan-cancer single-cell landscape.

**Supplementary Table S8.** Cellular communication results for patients BC_1790.

**Supplementary Table S9.** Cellular communication results for patients ICC_1012.

**Supplementary Table S10.** Cellular communication results for patients PDAC_5108.

**Supplementary Table S11.** Differentially expressed genes and cell type annotations for 23 clusters in all epithelial cells. Adjusted p values were calculated using two-sided Wilcoxon rank-sum test.

**Supplementary Table S12.** Differentially expressed genes and cell type annotations for 11 clusters in all stromal cells. Adjusted p values were calculated using two-sided Wilcoxon rank-sum test.

**Supplementary Table S13.** Differentially expressed genes and cell type annotations for 10 clusters in all endothelial cells. Adjusted p values were calculated using two-sided Wilcoxon rank-sum test.

**Supplementary Table S14.** Top 10 significant differential terms in each cell type of the single-lineage analyses.
